# Nitrogen Supply Affects Photosynthesis and Photoprotective Attributes During Drought-Induced Senescence in Quinoa

**DOI:** 10.3389/fpls.2018.00994

**Published:** 2018-07-30

**Authors:** Luisa Bascuñán-Godoy, Carolina Sanhueza, Cristián E. Hernández, Leonardo Cifuentes, Katherine Pinto, Rodrigo Álvarez, Marcia González-Teuber, León A. Bravo

**Affiliations:** ^1^Laboratorio de Fisiología Vegetal, Departamento de Botánica, Facultad de Ciencias Naturales y Oceanográficas, Universidad de Concepción, Concepción, Chile; ^2^Laboratorio de Ecología Evolutiva y Filoinformática, Departamento de Zoología, Facultad de Ciencias Naturales y Oceanográficas, Universidad de Concepción, Concepción, Chile; ^3^Centro de Estudios Avanzados en Zonas Áridas (CEAZA), La Serena, Chile; ^4^Escuela de Tecnología Médica, Facultad de Salud, Sede La Serena, Universidad Santo Tomas, La Serena, Chile; ^5^Laboratorio de Química Ecológica, Facultad de Química y Biología, Universidad de Santiago de Chile, Santiago, Chile; ^6^Instituto de Investigación Multidisciplinar en Ciencia y Tecnología, Universidad de La Serena, La Serena, Chile; ^7^Laboratorio de Fisiología y Biología Molecular Vegetal, Instituto de Agroindustria, Departamento de Ciencias Agronómicas y Recursos Naturales, Facultad de Ciencias Agropecuarias y Forestales, Center of Plant, Soil Interaction and Natural Resources Biotechnology, Scientific and Technological Bioresource Nucleus, Universidad de La Frontera, Temuco, Chile

**Keywords:** betacyanin, carotenoids, NPQ, thermal dissipation, lipid peroxidation, water scarcity, xanthophyll cycle, zeaxanthin

## Abstract

Drought during senescence has become more common in Mediterranean climates in recent years. *Chenopodium quinoa* Willd has been identified as tolerant to poor soil conditions and drought. Previous observations have found that sufficient nitrogen (N) supply mitigates yield losses under terminal drought conditions. However, there is no understanding of the mechanisms behind this effect. We hypothesized that N up-regulates both photosynthetic and photoprotective elements during drought-induced senescence, alleviating the negative impact of drought on yield. The role of N supply and terminal drought on photoprotection was tested using three Chilean quinoa genotypes from different climatic zones: Faro, UdeC9, and BO78. Plants were grown under high nitrogen (HN) or low nitrogen (LN) conditions and subjected to terminal drought at the onset of senescence. Photosynthetic and photochemical and non-photochemical processes were evaluated at both the onset of drought and after 15 days of drought conditions. N supplementation modified most of the physiological parameters related to photochemical dissipation of energy, photosynthesis, and yield in quinoa. In contrast, water restriction did not affect photosynthesis in quinoa, and its effect on yield was dependent on the genotype. A significant interaction N × G was observed in photosynthesis, relative water content, protein content, Fv/Fm, and chlorophylls. In general, Faro was able to maintain higher levels of these attributes under LN conditions than UdeC9 and BO78. In addition, the interacting effects of N × W regulated the level of most pigments in quinoa as well as the photoprotective induction of non-photochemical quenching (NPQ) during senescence. During terminal drought at LN conditions, Faro presented a larger NPQ induction under drought conditions than UdeC9 and BO78, which was supported by a larger zeaxanthin content and de-epoxidation state of the xanthophyll pool. Interestingly, BO78 did not induce NPQ in response to drought-induced senescence but instead enhanced the content of betacyanins. This response needs to be researched in future works. Finally, we observed that LN supply reduced the correlationship between the de-epoxidation state of the xanthophyll cycle and NPQ. This could be an indication that N supply not only compromised the capacity for photosynthetic performance in quinoa plants, but also affected the plasticity of thermal dissipation, restricting further changes during drought-induced senescence.

## Introduction

Plant leaf senescence can be triggered by normal developmental programming or suboptimal growth conditions. Physiologically, senescence is characterized by coordinated ultrastructural and metabolic changes accompanied by massive reprogramming of gene expression (Gan and Amasino, 1997). The most significant change is the breakdown of chloroplasts, which comprise more than 70% of nitrogen (N) taken up from the environment. Yield and seed/grain quality are dependent on this chloroplast breakdown and remobilization of nitrogen (Gregersen et al., 2013). Drought during leaf senescence leads to a decline in photosynthesis and production of assimilates. The consequence of decreased photosynthetic capacity during the grain filling period ultimately results in a decline in size and quantity of grain (Kichey et al., 2007; Gaju et al., 2011). In the last few decades, drought stress during senescence has become more common in Mediterranean-type environments affecting crop yields resulting in enormous economic losses (Ray et al., 2015).

Several studies have highlighted the influence of nitrogen supplementation on delayed senescence (Egli et al., 1976; Balazadeh et al., 2014), possibly as a result of a more robust photosynthetic apparatus capable of photochemically utilizing incident light (Balazadeh et al., 2014). Plants grown under limiting N conditions have a lower CO_2_ fixation capacity. Under high levels of light intensity, N-limited plants would have greater light energy excess (not used in photosynthesis) that could result in greater reactive oxygen species (ROS) production, triggering photoinhibition (Verhoeven et al., 1997). Thermal dissipation of excess absorbed energy at the antenna level is a rapid and efficient protective strategy to prevent over-reduction of the electron transport chain (Müller et al., 2001). Previous research has suggested that during down-regulation of PSII in senescent leaves, thermal dissipation and the xanthophyll cycle play an important role in photoprotection to prevent photoinhibitory damage (Lu et al., 2001; Yoo et al., 2003). Therefore, under conditions of limiting N when photochemical processes are extremely low, upregulation of thermal dissipation is expected. Nonetheless, published research from different plant species regarding N supply and photoprotection is contradictory. For example, N fertilization was observed to have no effect on major protective carotenoids and antioxidants in *Pinus radiata* (Posch et al., 2008). In contrast, high N supply in both *Coffea arabica* and wheat (Ramalho et al., 1998, 2000; Lu et al., 2003) revealed an up-regulation of xanthophyll cycle dependence and thermal energy dissipation. Furthermore, N fertilization can adequately protect *Coffea* plants against photodamage independently of the anticipated positive effects of N on photosynthetic capacity (Pompelli et al., 2010).

*Chenopodium quinoa* Willd (Chenopodiaceae) has attracted significant attention due to its remarkable high protein content and balanced essential amino acid composition (Thanapornpoonpong et al., 2008; Lutz and Bascunan-Godoy, 2017). Quinoa is a rustic crop that can grow in marginal conditions and for millennia has been cultivated organically, without the standard practice of fertilization and irrigation of traditional cereals. Its adaptability to unfavorable growth conditions has led it to be recognized as a strategic crop by the Food and Agricultural Organization (FAO) (FAO, 2011). Among the great number of quinoa ecotypes, coastal areas/lowlands are of particular importance as their photoperiod adaptation response makes them highly suitable for spreading quinoa cultivation into different climatic areas (Jacobsen and Stølen, 1993; Jacobsen, 1997; Bendevis et al., 2014). In fact, coastal/lowland Chilean genotypes have been used as parental elite sources in European quinoa breeding programs (Jacobsen and Stølen, 1993; Jacobsen, 1997). Chilean quinoa lowland genotypes present high phenotypic variability, agronomical performance, and tolerance to stress conditions (Berti et al., 2000; Ruiz-Carrasco et al., 2011; Bascuñán-Godoy et al., 2016; Morales et al., 2017; Gonzalez-Teuber et al., 2018).

One of the suggested strategies for improving quinoa crop yield is N fertilization leading to increased foliar area, thus improving light interception and photosynthesis (Razzaghi et al., 2012). In water-stressed quinoa, N confers a certain degree of tolerance-regulating abscisic acid (ABA) concentration and stomatal closure (Alandia et al., 2016). However, the physiological mechanisms for coping with drought under limiting N supplementation have garnered little attention. Further, the effect of N supply on photoprotective mechanisms and their relationship with grain yield is still unclear.

The aim of this work was to understand how N supply regulates the mechanisms of photoprotection, especially thermal dissipation, during drought-induced senescence progress using three different quinoa lowland Chilean genotypes. We hypothesized that N up-regulates both photosynthetic capacity and photoprotective attributes during senescence under terminal drought. The role of the xanthophyll cycle and thermal dissipation during drought-induced senescence were also investigated.

## Materials and Methods

### Plant Material and Growth Conditions

Experiments were conducted from September 2015 until February 2016 in a greenhouse located at La Serena University (29.54° S, 71.14° E). The greenhouse was provided with approximately 1,200 μmol m^-2^ s^-1^ PAR at noon (natural light), and at maximum and minimum temperatures (daily ranges) of 23°C and 17°C, respectively, 12 h day length, and 80% relative humidity.

### Experimental Design

The experiment was run as a completely randomized design with four factors under study: genotypes, N treatment, water treatment and time. We used supplementary plants to prevent bordering effect.

#### Genotypes (G)

Three Chilean coastal/lowland genotypes of *Chenopodium quinoa* Willd (Chenopodiaceae) with similar morphological and phenological characteristics but from different geographic and climatic areas were utilized. Faro seeds were obtained from the Cooperative Las Nieves, while UdeC9 and BO78 seeds were obtained from the National Seed Bank collection at Vicuña, Chile (INIA-Intihuasi). Seeds were collected for the genotypes from the following locations: Faro (34.65° S, 71.91° E), UdeC9 (37.65° S, 71.6° E), and BO78 (38.51° S, 71.4° E).

Seeds of different genotypes were germinated directly in soil in 10 L pots (22 cm tall by 28 cm diameter). The pots were filled with equal amounts of dry soil (5 kg). The soil consisted of a mixture of 80% sand, 20% peat, and a basal fertilization of N: 40 mg/kg, P: 96 mg/kg, and K: 690 mg/kg. The soil had volumetric soil water content (%, vol.) of 30% at 100% pot water holding capacity (WHC). Before the establishment of water regimes, all plants were fully irrigated to WHC.

#### Nitrogen Treatment (N)

Soils were supplemented with urea to reach two N levels, high nitrogen (HN; 0.6 g of N per pot) and low nitrogen (LN; 0.30 g of N per pot). No additional N was applied during the experiment. Sixteen plants (one per pot) of each genotype and N regime were maintained at optimal soil moisture levels (100% WHC) by irrigating with 1 L of water every 3 days.

#### Water Treatment (W)

Fourteen days after flowering (DAF), the HN and LN experimental plants were sub-divided into control (C) and drought-stressed (S) leading to four treatment groups: HNC (high nitrogen supply, control 100% WHC), LNC (low nitrogen supply, control 100% WHC), HNS (high nitrogen supply, drought conditions), and low nitrogen stress (LNS) (low nitrogen supply, drought conditions). The drought treatment was applied by reducing irrigation by 70% (300 mL) relative to control plants (1 L) for the remaining life cycle. Throughout the experiment, the soil humidity was monitored and maintained at 100% of WHC in control pots and 30% of WHC in water-stressed replicates (eight pots per treatment). The water content of each pot was monitored according to weight every 3 days, and additional irrigation was applied to fulfill humidity targets. We used four pots for biomass analysis and four for physiological measurements.

#### Time (T)

Photosynthesis, chlorophyll fluorescence parameters, and pigments were evaluated at the onset of the treatment (day 0) and 15 days post-treatment (day 15). These times which corresponded to 14 and 29 DAF represent the onset (panicle development) and advancement of senescence (pigments shift), respectively, in quinoa.

### Variables Measured

#### Biomass and Grain Yield

To evaluate the effect of N on biomass production, aerial parts and roots dry mass were determined at the beginning of the drought treatment (day 0). Tissues were initially dried at 80°C over a period of 3 h, followed by drying at 60°C until constant weight was achieved (*n* = 4).

Grain yield for all treatments was determined at the end of the growth season (180 days after sowing) based on the total weight production of each plant (*n* = 4).

#### Gas Exchange Measurements

Gas-exchange measurements were conducted in fully expanded leaves (third leaf from the top) from each group (*n* = 4) using LI-COR 6400-40Li-6400 (Li-Cor Inc., Nebraska, United States). Leaves were first equilibrated at a photon density flux of 1500 μmol m^2^s^-1^ for at least 10 min and 370 μmol mol^-1^ of external CO_2_. Leaf temperature was maintained at 28°C, and the leaf-to-air vapor pressure deficit was between 1 kPa and 1.3 kPa. These conditions were kept constant for the determination of CO_2_ assimilation rate (*P_n_*) and stomatal conductance (*g_s_*). The intrinsic water use efficiency (_i_WUE) was calculated as the ratio of net CO_2_ assimilation to stomatal conductance for water vapor.

#### Protein, Relative Water Content, and Lipid Peroxidation in Leaves

Samples were taken at 12 p.m. and leaf relative water content (RWC) was calculated as follows: RWC = (fresh weight - dry weight)/(turgid weight - dry weight) × 100. Turgid leaf weight was determined after keeping the leaf in distilled water in darkness at 4°C to minimize respiration loss until a constant weight was achieved (full turgor, typically after 12 h).

Lipid peroxidation in leaves of plants from each group (*n* = 4) was determined *in vitro* by estimating the formation of malondialdehyde (MDA) according to the method described by Ortega-Villasante et al. (2005). Frozen leaf tissue (0.1–0.2 g) was homogenized in 1 mL of TCA–TBA–HCl reagent [15% (w/v)] trichloroacetic acid, 0.37% (w/v) 2-thiobarbituric acid, 0.25 M HCl, and 0.01% butylated hydroxytoluene. After homogenization, samples were incubated at 90°C for 30 min and centrifuged at 12,000 × *g* for 10 min. Absorbance was measured at 535 nm and 600 nm. Bradford Assay (Bradford, 1976) was used for protein quantification in leaves using BSA as the standard protein (*n* = 4).

#### Chlorophyll Fluorescence Measurements

Chlorophyll (Chl) fluorescence measurements were performed using a portable fluorimeter (FMS 2, Hansatech Instruments Ltd., Norfolk, United Kingdom). Leaves of plants from each group (*n* = 4) were dark-adapted at ambient temperature for 30 min prior to measurement. Actinic light for measurements was of 1,200 μmol photons m^-2^ s^-1^ following Bascuñán-Godoy et al. (2015). Fluorescence parameters were calculated as described in Maxwell and Johnson (2000) and Kramer et al. (2004).

#### Betacyanins Analysis

Betacyanins were extracted in water and pigment content determined by spectrophotometrical determination at 536 nm with an Infinite 200 Pro spectrophotometer (Tecan, Männedorf, Switzerland). The betacyanin content of the plant aqueous extracts was estimated using the molar extinction coefficient for amaranthine and an molecular weight (MW) of 726.6 (*n* = 4).

#### Pigments and Xanthophyll Cycle Analyses

Fresh leaf tissue (100 mg) from different genotypes, treatments, and experimental time points (*n* = 4) was collected, flash frozen in liquid nitrogen, and stored at -80°C. Subsequently, samples were ground into powder using liquid N combined with a spatula tip of CaCO_3_, and lyophilized at low temperature before analysis. Pigments were extracted with 1 mL 100% high performance liquid chromatography (HPLC)-grade acetone at 4°C under dim light. The extract was clarified by centrifugation and the supernatant was filtered through 0.45 μm syringe filter. Pigments chosen for analysis included chlorophyll *a* and *b*, β-carotene (β-car), neoxanthin, violaxanthin, antheraxanthin, and zeaxanthin. Pigments were measured using the HPLC method described by García-Plazaola and Becerril (1999) with modifications included in Sáez et al. (2013). De-epoxidation state (DEPS) of the xanthophyll pool was calculated as follow: DEPS = (V + 0.5A)/(V + A + Z), where V is violaxanthin, A is antheraxanthin, and Z is zeaxanthin.

### Statistical Analysis

A four-way analysis of variance (ANOVA) was conducted in order to determine the effects of genotypes (Faro, UdeC9, BO78), N treatments (HN, LN), water treatments (C, S) and time points (day 0, day 15) on pigments and physiological parameters. A Newman–Keuls method (level of significance *p* < 0.05) was used as a *post hoc* test in order to reveal significant differences among groups. Assumptions of normality and homogeneity of variance were tested using the Kolmogorov–Smirnov and Lilliefors tests. All statistical analyses were performed using STATISTICA 6.0 software (Statsoft Inc., version 6.0^1^).

Linear Pearson’s correlation coefficient (*r*) was used to examine the correlations between yield and photosynthesis, changes in photosynthesis, and thermal dissipation [non-photochemical quenching (NPQ)], and NPQ and de-epoxidation state of the xanthophylls cycle (DEPS).

## Results

### Effect of Genotype Treatments and Time on Photosynthetic Traits

**FIGURE 1:**
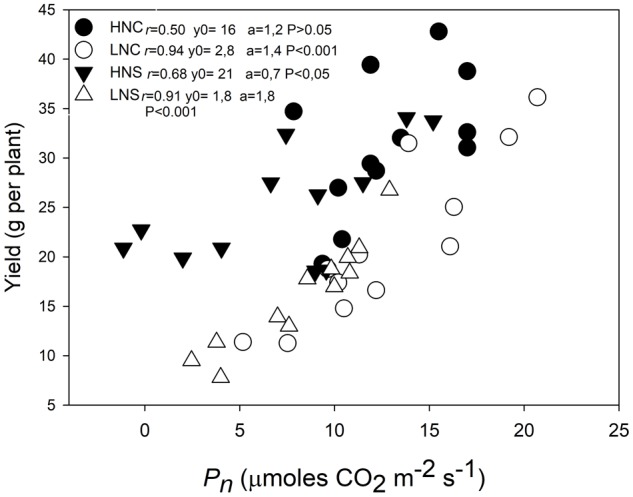
Relationship between grain yield and photosynthesis in three quinoa genotypes subjected to different regimens of nitrogen and water stress. The photosynthesis measurements were performed in fully expanded leaves (third leaf from the top) in plants at 15 days after water treatment imposition in plants at HNC, HNS, LNC, and LNS treatments. Symbols represent treatments: HNC (black circles), LNC (empty circles), HNS (black down triangles), and LNS (empty up triangles). Linear Pearson’s correlation coefficient (*r*) was used to examine the correlations.

The regression plot of grain weight per plant against photosynthesis indicated a positive relationship and a high grade of linearity (**Figure 1**). Plant yield under LN treatment (LNC and LNS) showed a stronger and significant correlation with *P_n_* (*r* > 0.9; *p* < 0.001) than under HN treatment (HNC with *p* > 0005 and *r* = 0.5; and HNS with *p* < 0.05 and *r* = 0.68). Regarding water conditions the correlation between yield and *P_n_* was only statistically significant at HNS (*p* < 0.05) but not at HNC conditions (*p* > 0.05). At LNC and LNS conditions correlations between yield and *P_n_* were statistically significant (*p* = 0.001). However, grain yield was consistently higher in HN than in LN even at similar levels of *P_n_*.

**FIGURE 2:**
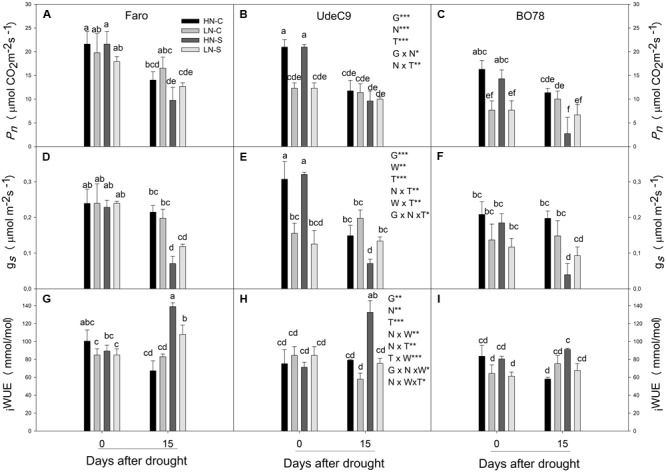
Effect of N supplementation and water stress treatment on photosynthetic parameters. Net phosynthesis **(A–C)**; conductivity (*g*_s_) **(D–F)**, intrinsic water use efficiency (_i_WUE) **(G–I)** were taken in well-developed leaves at mid-morning at both 0 and 15 days after drought. Bars represent treatments: HNC, LNC, HNS, and LNS. Values are mean ± SE (*n* = 4). Different letters represent significant differences among genotypes, N supply, water treatment, and time (*p* < 0.05) using four-way analysis of variance (ANOVA). Newman–Keuls was used as a post hoc test.

The four-way ANOVA of photosynthetic rate (*P_n_*) revealed a significant effect of interactions G × N (*p* = 0.041) and N × T (*p* = 0.002) (**Table 1**). At day 0, three genotypes exhibited similar *P_n_* rates at HN (HNC and HNS plants) conditions (**Figures 2A–C**), however, genotypes showed a differential performance under LNC conditions. At both times (0 and 15 days) Faro showed significantly higher *P_n_* than UdeC9 and BO78 at LNC (G × N) (**Figures 2A–C**). Over time greater reductions of *P_n_* were observed in plants developed at HNC in comparison to LNC conditions (N × T). However, drought-stressed plants (HNS and LNS) maintained similar *P_n_* values than their respective controls (**Figures 2D–F**).

**Table 1:** *P-*values for the effects of G, N, W, T and their interactions determined by four-way ANOVA analysis on physiological attributes: Net photosynthetic rate (*P_n_*), stomatal conductance (*g_s_*), intrinsic water-use efficiency (_i_WUE), relative water content of leaves, proteins, lipid peroxidation (estimated by MDA), the maximal efficiency of PSII (Fv/Fm), quantum yield of PSII (ΦPSII) and the non-photochemical quenching (NPQ) in three genotypes of *Chenopodium quinoa* grown at two levels of nitrogen supplementation and exposed to 15 days of drought during senescence.

	G	N	W	T	G × N	G × W	G × T	N × W	N × T	T × W	G × N × W	G × N × T	G × W × T	N × W × T	G × N × W × T
*P_n_*	**0.000**	**0.005**	0.107	**0.000**	**0.041**	0.503	0.753	0.425	**0.002**	0.107	0.660	0.860	0.503	0.425	0.660
*g_s_*	**0.038**	0.140	**0.002**	**0.000**	0.224	0.843	0.670	0.281	**0.004**	**0.002**	0.787	**0.017**	0.843	0.281	0.787
_i_WUE	**0.003**	**0.002**	0.444	**0.000**	0.942	0.581	0.684	**0.045**	**0.011**	**0.000**	**0.031**	0.912	0.684	**0.011**	0.912
RWC	**0.000**	**0.035**	**0.000**	**0.000**	**0.007**	**0.035**	0.465	0.758	0.616	**0.000**	0.439	0.584	**0.001**	0.172	0.122
Proteins	**0.000**	**0.000**	0.217	**0.000**	**0.027**	**0.015**	**0.000**	0.777	**0.005**	0.217	0.880	0.241	**0.015**	0.777	0.880
MDA	**0.024**	0.224	**0.033**	**0.000**	0.354	0.173	**0.004**	0.531	0.429	**0.033**	0.271	0.761	0.173	0.531	0.271
Fv/Fm	0.696	**0.000**	**0.001**	0.823	**0.025**	0.357	**0.001**	0.831	**0.016**	0.823	0.289	0.748	**0.001**	**0.016**	0.748
ΦPSII	**0.000**	**0.000**	0.683	**0.000**	0.971	0.909	**0.039**	0.588	**0.000**	0.683	0.692	0.127	0.909	0.588	0.692
NPQ	**0.000**	**0.000**	**0.000**	**0.032**	0.862	**0.000**	**0.040**	**0.000**	0.923	**0.032**	0.208	0.738	**0.040**	0.923	0.738

*Significant *P-*values are in bold and red. The underline shows the significant independent effect that are not included in a significant interaction, and the more complex interaction that includes the effect of independent factors or more simple interactions (The posterior interpretation is only based on the complex interactions that nests the simple effect, and independent effect that are not included in an interaction). (See **Supplementary Table S1** for the four-way ANOVA details).*

Stomatal conductivity (*g_s_*) was significantly affected by interactions T × W (*p* = 0.002) and G × N × T (*p* = 0.017) (**Table 1**). A differential effect of water between times was observed (T × W), and also a differential effect of nitrogen among genotypes over time (G × N × T). At the beginning of the experiment (day 0), LN supplied plants (LNC and LNS) of UdeC9 showed 40% lower *g_s_* than HN supplied plants (**Figure 2E**). This response contrasted with Faro and BO78, which maintained similar *g_s_* levels within the N treatment (**Figures 2D,F**). A reduction of *g_s_* levels was observed in HNS and LNS in comparison to their controls for all genotypes (G × N × T).

Interestingly, _i_WUE was dependent on two complex interactions: G × N × W (*p* = 0.031) and N × W × T (*p* = 0.011) (**Table 1**). N and W significantly affected _i_WUE changes over time (N × T × W) and among genotypes (G × N × W) (**Table 1**). During the progress of drought, plants developed at HNS but not at LNS conditions significantly increased _i_WUE in comparison to their controls (**Figures 2G–I**). Only under drought conditions Faro and UdeC9 significantly decreased _i_WUE in response to LN. In contrast, B078 maintained similar values of _i_WUE independent of drought and the nitrogen treatment (G × N × W).

### Changes in Protein Amount, RWC, and Lipid Peroxidation in Response to N-Water Condition Treatments

**FIGURE 3:**
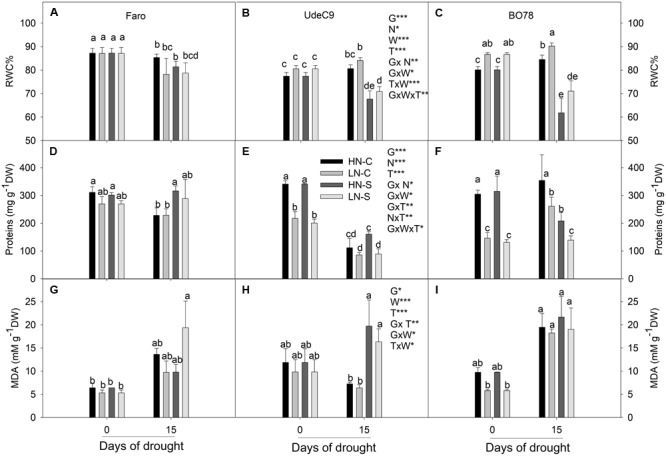
Relative water content (RWC) **(A–C)**, protein content **(D–F)**, malonaldehide content (MDA) **(G–I)** changes under different treatments of N supplementation and water stress during senescence. Results were calculated from four independent measurements of different plants. Leaf samples were taken at mid-morning at both 0 and 15 days after drought. Bars show mean values ± SE (*n* = 4). Different letters represent significant differences genotypes, N supply, water treatment, and time (*p* < 0.05) using four-way ANOVA. Newman–Keuls was used as a post hoc test.

Interactions G × N (*p* = 0.007) and G × W × T (*p* = 0.001) significantly affected RWC of quinoa plants (**Table 1**). At time 0, N differentially affected RWC among genotypes (G × N) (**Figures 3A–C**). BO78 showed higher RWC at LNC conditions than at HNC, which contrasted with that observed for Faro and UdeC9 that presented similar levels of RWC between nitrogen treatments. Fifteen days of drought induced significant reductions of RWC in UdeC9 and BO78. Faro was able to maintain RWC, exhibiting higher levels, at both HNS and LNS conditions, in comparison to other genotypes (G × W × T).

Protein content was significantly affected by interactions G × N (*p* = 0.027), N × T (*p* = 0.005), and G × W × T (*p* = 0.015) (**Table 1**). At time 0, there was a notorious effect of N on the protein content among genotypes (G × N) (**Figures 3D–F**). Whereas Faro showed similar levels of proteins at both HN (C and S) and LN (C and S) conditions, Udec9 and B078 showed significant reductions of the protein content in response to LN conditions. Time differentially affected the protein content of HN and LN plants. HN plants showed a more notorious reduction of the protein content after 15 days in comparison to LN plants, whose protein content was maintained over time (N × T). Fifteen days of drought differentially affected the protein content among genotypes, with Faro showing the highest protein content at both HNS and LNS conditions (G × T × W) (**Figure 3D**).

The level of lipid peroxidation (MDA content) was significantly affected by interactions G × T (*p* = 0.004) and W × T (*p* = 0.033) (**Table 1**). Over time, changes in MDA levels were dependent on the genotype (G × T). Whereas Faro and B078 reached similar contents of MDA, independent of the water treatment (G × T), UdeC9 significantly increased MDA levels in response to drought (**Figures 3G–I**).

### Changes in Fluorescence of Chlorophyll *a* Parameters During Senescence

**FIGURE 4:**
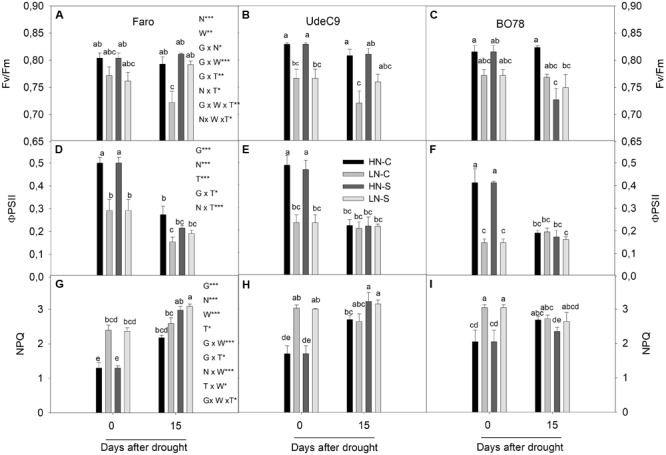
Photochemical and non-photochemical fluorescence parameters during grain filling in three genotypes of quinoa exposed to water stress at two levels of N supplementation. Fv/Fm **(A–C)** is the maximal efficiency of PSII, ϕPSII **(D–F)** is the quantum yield of PSII and NPQ **(G–I)** is the non-photochemical quenching. Results were calculated from four independent measurements of different individuals. Bars show mean values ± SE (*n* = 4). Different letters represent significant differences between genotypes, N supply, water treatment, and time (*p* < 0.05) using a four-way ANOVA. Newman–Keuls was used as a post hoc test.

Maximum quantum efficiency (Fv/Fm) was significantly affected by interactions G × N (*p* = 0.025), G × W × T (*p* = 0.001), and N × W × T (*p* = 0.016) (**Table 1**). Nitrogen differentially affected Fv/Fm among genotypes (G × N) (**Figures 4A–C**). Whereas LN reduced Fv/Fm for UdeC9, Fv/Fm tended to be similar between both nitrogen treatments for Faro and B078. After 15 days of drought Fv/Fm maintenance was different among genotypes and watering conditions (G × W × T) (**Figures 4A–C**). At that time, only Faro tended to increase Fv/Fm values in response to drought. In addition, the significant interaction N × W × T revealed that after 15 days of drought, differences in Fv/Fm induced by N vanished because of the drought (**Figures 4A–C**).

The use of energy (quantum yield of PSII) was significantly affected by interactions G × T (*p* = 0.039) and N × T (*p* < 0.001) (**Table 1**). PSII was greater for Faro and for UdeC9 in comparison to BO78, but only at time 0 (G × T, *p* < 0.05). At that time, ϕPSII was significantly higher at HN conditions (HNC and HNS) in comparison to LN conditions (LNC and LNS) for all genotypes (N × T) (**Figures 4D–F**). Nevertheless, at 15 days’ time stronger reductions of ϕPSII were observed for all genotypes, independent of the nitrogen treatment (N × T) (**Figures 4D–F**).

Thermal dissipation, measured as NPQ, was significantly affected by interactions N × W (*p* < 0.0001) and G × W × T (*p* = 0.040) (**Table 1**). Nitrogen differentially affected NPQ levels depending on water (N × W). Over time drought differentially affected NPQ levels among genotypes. At time 15, Faro and UdeC9 increased NPQ levels in response to drought, whereas BO78 tended to maintain its NPQ levels (G × T × W) (**Figures 4G–I**).

**FIGURE 5:**
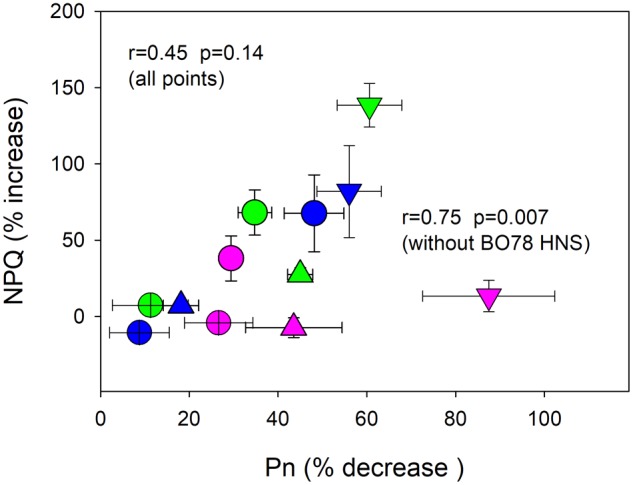
Relationship between percent decrease in photosynthesis and increase of non-photochemical quenching (NPQ). Values were calculated as percent of change of plants at 29 DAF relative to initial control plant values (at 14 DAF). Symbols represent treatments HNC (circles), LNC (crossed circles), HNS (down triangles), LNS (up triangles), and color represents genotypes Faro (green), UdeC9 (blue), and BO78 (pink). Mean values ± SE were calculated from four independent measurements. Linear Pearson’s correlation coefficient (*r*) was used to examine the correlations.

Complementarily, the relationship between NPQ level (%) and *P_n_* changes (%), at times 0 and 15, was studied (**Figure 5**). Most plants experienced an increase in NPQ concomitant with a decrease in photosynthesis due to senescence and drought-induced senescence (**Figure 5**). Nevertheless, BO78, which displayed the largest reduction in *P_n_* at HNS, did not show changes in the percentage of NPQ. The *r* value for the relationship between NPQ enhancement (%) and *P_n_* reduction (%) was 0.45 (*p* = 0.14). However, by omitting this group (BO78 at HNS, plotted with pink triangles), the relation was improved to *r* = 0.75 (*p* = 0.007).

### Pigments Changes During Senescence in Differentially Supplied Plants of Quinoa

The four-way ANOVA revealed that the interaction N × W had a significant effect on most studied pigments (**Table 2** and **Supplementary Tables S2, S3**), including Chl *a* and *b*, violaxanthin, antheraxanthin, neoxanthin, lutein, and β-carotene. This means that most pigments are highly dependent on watering conditions and nitrogen levels. Both Chls *a* and *b* were significantly affected by interactions G × N (*p* = 0.019) and N × W (*p* = 0.001). At day 0, both HNC and HNS plants of Faro and UdeC9 showed similar Chls contents; nevertheless, at LN UdeC9 significantly reduced its Chl content in comparison to Faro (G × N). Our analysis showed that drought differentially affected the Chl content between nitrogen treatments (N × W). In general, the lowest contents of Chl were observed in LNS plants.

**Table 2:** *P-*values for the effects of G, N, W, T and their interactions determined by four-way ANOVA analysis on chlorophylls, xanthophylls, de-epoxidation state of xanthophyll cycle (DEPS), carotenoids, and betacyanins in three genotypes of *Chenopodium quinoa* grown at two levels of nitrogen supplementation and exposed to 15 days of drought.

	G	N	W	T	G × N	G × W	G × T	N × W	N × T	T × W	G × N × W	G × N × T	G × W × T	N × W × T	G × N × W × T
Chlorophyll *a*	**0.000**	**0.000**	**0.000**	0.862	**0.019**	0.280	0.503	**0.001**	0.988	0.862	0.232	0.841	0.503	0.988	0.841
Chlorophyll *b*	**0.000**	**0.000**	**0.000**	0.798	**0.049**	0.110	0.615	**0.001**	0.927	0.798	0.305	0.884	0.615	0.927	0.884
Violaxanthin	**0.012**	**0.000**	**0.000**	0.219	0.060	0.788	0.928	**0.011**	0.935	0.219	0.093	0.891	0.928	0.935	0.891
Anteraxanthin	0.639	**0.002**	0.082	**0.035**	0.233	**0.002**	0.659	**0.011**	0.726	**0.035**	0.173	0.859	0.659	0.726	0.859
Zeaxanthin	**0.002**	**0.003**	**0.000**	**0.000**	0.817	**0.000**	**0.001**	0.573	0.225	**0.000**	**0.019**	0.328	**0.001**	0.225	0.328
Xanthophylls pool	**0.007**	**0.017**	**0.000**	0.932	0.094	0.446	0.845	0.091	0.728	0.932	0.381	0.840	0.845	0.728	0.840
DEPS	0.323	**0.000**	**0.000**	**0.000**	**0.041**	**0.001**	**0.009**	**0.005**	0.165	**0.000**	**0.000**	0.189	**0.009**	0.165	0.189
Neoxanthin	**0.000**	**0.000**	**0.000**	0.713	0.065	0.330	0.684	**0.001**	0.933	0.713	0.281	0.924	0.684	0.933	0.924
Lutein	**0.000**	**0.000**	**0.000**	0.658	0.121	0.224	0.788	**0.010**	0.887	0.658	0.256	0.868	0.788	0.887	0.868
β-Carotene	**0.001**	**0.000**	**0.000**	0.829	0.052	0.423	0.593	**0.002**	0.890	0.829	0.273	0.882	0.593	0.890	0.882
Betacyanin	**0.000**	0.262	**0.000**	**0.000**	**0.005**	**0.000**	**0.000**	**0.002**	**0.000**	**0.000**	**0.000**	**0.000**	**0.000**	**0.000**	**0.000**

*Significant *P-*values are in bold and red. The underline shows the significant independent effect that are not included in a significant interaction, and the more complex interaction that includes the effect of independent factors or more simple interactions (The posterior interpretation is only based on the complex interactions that nests the simple effect, and independent effect that are not included in an interaction). (See **Supplementary Table S3** for the four-way ANOVA details).*

Betacyanins were significantly affected by interactions of all factors (**Table 2** and **Supplementary Table S3**). At time 0, BO78 under both HN and LN conditions showed higher levels of betacyanin than Faro and UdeC9 (**Supplementary Table S2**). No differences were observed by N supply as a single factor, however all their interactions were significant. Time, however, induced significant increases in betacyanins for all genotypes; nevertheless, it was remarkably higher for BO78. Whereas at HNS conditions (25 μg/100 g DW) BO78 reached 2.5 times more betacyanins than at HNC conditions (10 μg/100 g DW), similar levels of betacyanins were observed between LNS and LNC. After the drought period, in general the highest levels of betacyanins in HNS plants were observed for B078 (G × N × W × T).

Total xanthophyll pool size was significantly affected by G (*p* < 0.01), N (*p* < 0.02), and W (*p* < 0.001) (**Table 2** and **Supplementary Table S3**). In general, LN and drought reduced the xanthophyll pool size (**Supplementary Table S2**). Faro tended to maintain a higher xanthophyll pool with regards to UdeC9 and BO78. The de-epoxidation state of the xanthophyll pool (DEPS) was significantly affected by interactions G × N × W (*p* < 0.001) and G × W × T (*p* < 0.01). Over time, changes in DEPS were more pronounced in Faro than in UdeC9 and BO78 (G × W × T). In addition, the DEPS levels reached in response to drought were dependent on the genotype and the nitrogen treatment (G × N × W). Higher DEPS levels were observed at LNS than at HNS for Faro and for UdeC9; however, BO78 showed similar DEPS levels at both N conditions. The highest DEPS was observed for Faro at LNS conditions.

Drought, as a single factor, significantly affected the amount of carotenoids, with the exception of antheraxanthin (**Table 2**). Both, DEPS and zeaxanthin increased in response to drought (**Supplementary Table S2**). These changes were concomitant with reductions in violaxanthin, and in neoxanthin, lutein, and β-carotene for UdeC9 and BO78. Greater contents of DEPS, zeaxanthin, and antheraxanthin were observed for Faro in comparison to UdeC9 and BO78 (**Supplementary Table S2**).

**FIGURE 6:**
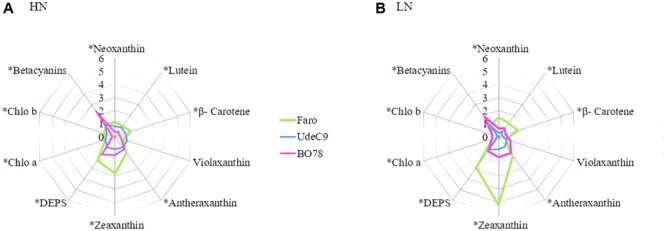
Effect of water stress during senescence on carotenoids, chlorophylls, and betalains in three genotypes of quinoa grown at two levels of nitrogen supplementation. Radar charts showing changes in the relative contents of pigments in three genotypes of *Chenopodium quinoa* grown at HN **(A)** and LN **(B)** subjected by 15 days to water stress respect to control plants. Relative contents were calculated as the ratio of the relative content for each pigment to their control sample. Values are means of *n* = 4. Asterisks indicate significant differences among genotypes (*p* < 0.05) using a one-way ANOVA.

For better visualization of drought effects on individual pigments under both N conditions, the relative pigment content was calculated as the ratio of each pigment under water deficit to its control condition: HNS:HNC and LNS:LNC (**Figure 6**). Carotenoids, zeaxanthin, and antheraxanthin showed the most evident increase in *C. quinoa* (at both HNS and LNS relative to controls). However, lutein, neoxanthin, and β-carotene showed significant differences among genotypes. Faro exhibited the largest changes in carotenoid levels and in DEPS under both HN and LN conditions, whereas the UdeC9 and BO78 genotypes remained fairly stable. This latter genotype (BO78) showed a considerable accumulation of betacyanins in response to drought; this was neither observed for Faro nor for UdeC9. BO78 exhibited a 2–2.5 times increase of betacyanin levels at LNS and HNS with respect to controls, LNC and HNC (**Figure 6**).

**FIGURE 7:**
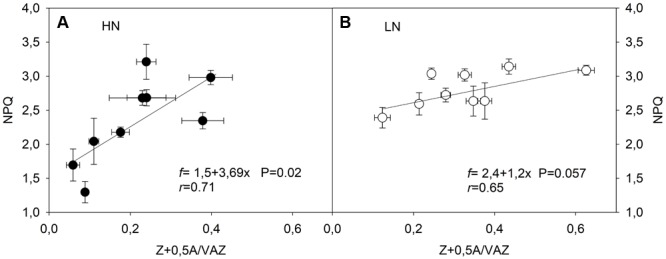
Relationship between the de-epoxidation state of xanthophyll pool (DEPS) and non-photochemical quenching (NPQ) at HN **(A)** and LN **(B)** supplementation in three genotypes of *C. quinoa*. The pigments analyses and NPQ determinations were performed in fully expanded leaves (third leaf from the top) from plants subjected to all treatments and times. Values were calculated as mean values ± SE from four independent plants. Linear Pearson’s correlation coefficient (*r*) was used to examine the correlations. Kolmogorov–Smirnov and Lilliefors test was used for normality analysis.

In order to investigate the role of N supply on thermal dissipation ability, the relationship between DEPS changes and NPQ was evaluated. This relationship was positive and dependent on the N level only at HN (slope = 3.7; *p* = 0.02) (**Figure 7**). Further, a significant relationship was observed at HN (*p* = 0.02), but not at LN conditions (*p* = 0.057). This response might indicate that subtle changes in DEPS induced greater NPQ changes at HN than at LN conditions.

## Discussion

N metabolism plays a critical role in the maintenance of photosynthesis as measured by net photosynthetic rates (*P_n_*) and grain yield in quinoa (Bascuñán-Godoy et al., 2016). To examine the effect of N supply and terminal drought on quinoa, three geographically different cultivars were chosen for these experiments. In this research we examined the effect of N supply on both the maintenance of photosynthesis and the induction of pigment readjustment during drought-induced senescence.

### N More Than Water Affected the Relationship of Photosynthesis and Yield in Quinoa

It has been reported that the availability of N delays leaf senescence and leads to increased grain yield in many species (Egli et al., 1976; Maxclaux-Daubresse et al., 2001). Quinoa exhibited a positive relationship between grain yield and photosynthesis (**Figure 1**). N supply strongly affected the relationship between photosynthesis and yield, as at similar *P_n_* the HN (both HNC and HNS) plants generated higher yields than the LN (LNC and LNS) plants. Water stress negatively effected grain yield significantly (**Supplementary Table S1**), but not *P_n_* (**Figures 2A–C** and **Table 1**) and there was no significant interaction between N × W observed with either *P_n_* or yield (**Table 1**). Drought had a more subtle effect than N on their relationship (**Figure 1**). It was remarkable that yield in LN supplied plants (LNC or LNS) (*p* < 0.001) and HNS (*p* < 0.05) showed less data dispersion relative to those under HNC conditions (*p* > 0.05). This may imply that yield was more dependent of the maintenance of *P_n_* at LN and drought conditions than at HN and well water supplied conditions. This could be explained because under sufficient N conditions, processes other than *P_n_* become relevant to grain yield, such as the remobilization and translocations of nutrients to seeds (Maxclaux-Daubresse et al., 2001; Masclaux-Daubresse et al., 2010).

### The N Effect on Photosynthetic Traits Changed Trough the Senescence Progress

Variation in *P_n_* between genotypes’ treatments and time (**Figures 2A–C**) were closely related to changes in stomata conductivity (*g_s_*) (**Figures 2D–F**). N affected differentially *P_n_* and *g_s_* depending on genotype and day of measurement. At the onset of treatments [day 0, 14 DAF], LN (in comparison with HN) conditions had enormously compromised *P_n_* and *g_s_* of BO78 and UdeC9, but not of Faro. Examination of both the plant biomass (**Supplementary Figure S1**) and protein content (**Figures 3D–F**) showed that BO78 and UdeC9 were compromised greatly at LN conditions, suggesting a higher sensitivity to insufficient N in these genotypes compared with Faro. Faro, the most northern genotype, typically grows under poorer soils with dryer conditions than the mid-range UdeC9 and southern BO78. Faro has high N use efficiency capacity (Berti et al., 2000) and greater drought tolerance with respect to the BO78 genotype (Bascuñán-Godoy et al., 2016). These characteristics could be behind the remarkable photosynthetic performance of Faro during terminal drought under LN conditions.

During the progress of senescence (15 days after water treatment) stronger reductions were observed in *g_s_* and *P_n_* in plants under HNS than LNS conditions (**Figures 2A–C** and **Table 1**). Interestingly, the _i_WUE measuring photosynthetic water usage was affected significantly with N and W treatment combinations during the progress of senescence. Our results are consistent with those previously reported for quinoa (Alandia et al., 2016), which indicate that plants at LNS display poorer control over water loss than those under HNS conditions. However, we found that water control responses were also dependent on genotype, showing Faro and UdeC9 the higher values of _i_WUE than the southern genotype (BO78).

The results were supported by the RWC measurements (**Figures 3A–C**). There was a statistically significant impact of nitrogen and water treatment on genotypes resulting in different RWC values (**Table 1**). Further, the maintenance of RWC through the senescence progress depended on genotype (**Table 1**). At both water stress conditions (HNS and LNS), Faro presented the highest maintenance of RWC suggesting a larger water economy relative to the UdeC9 and BO78 genotypes (**Figure 3**). When examining the root mass accumulated by the different genotypes, Faro had larger root development at LN conditions which could increase water and nutrient uptake from soil contributing to its maintenance of RWC (**Supplementary Figure S1**). The increased root mass characteristic of Faro also could be responsible for the larger protein level in leaves (by changes in the ability for N uptake) compared with UdeC9 and BO78, under LN conditions (**Figures 3D–F**). As RuBisCO and thylakoid proteins are the principal form of N that is stored in leaves (Marschner, 2012) the changes in these protein levels may be strongly influencing changes in *P_n_* and *g_s_*. The strong reduction of proteins through the senescence process observed in UdeC9 and BO78 contrasted with the maintenance of protein level in Faro. The signals responsible for Faro’s maintenance of protein content resulting in delay senescence are unknown, however, increased production of cytokinins with respect to ABA have been demonstrated in rice (Reguera et al., 2013).

The expansion of ROS that negatively impact lipids and proteins in chloroplasts is one of the oldest and most popular theories concerning development of senescence (Wang et al., 2013). However, MDA (used as estimation of lipid peroxidation) was not affected by N treatments (**Table 1** and **Figures 3G–I**). Perhaps quinoa plants developed at LN conditions (with poor investment in photosystem proteins) would have reduced light energy capture capacity, thus a lower probability of producing ROS. Nevertheless during senescence progress terminal drought affected significant MDA content in quinoa leaves (**Figures 3G–I**). Coincidentally, water and time interaction (**Table 1**) significantly impacted *g_s_*, which would result in reduced gas exchange and CO_2_ fixation from the Calvin cycle. Decreased carbon fixation would reduce the oxidation of NADP^+^ necessary to recycle it back to acquire electrons from the photosynthetic electron transport chain, thus increasing the transport of electrons to oxygen-producing ROS. More efforts are necessary to understand the development of ROS and antioxidant mechanisms that regulate ROS production in quinoa under different conditions of water and N supply.

### N but Not Drought Regulated the Photochemical Processes, While the Interaction of N and Drought Exacerbated Thermal Dissipation During Senescence

For a deeper understanding of energy use/dissipation in quinoa, fluorescence parameters were evaluated. N supply affected all fluorescence parameters measured and different interactions among factors were observed according to the parameter studied (**Table 1**). The Fv/Fm ratio, which reflects the maximum quantum efficiency of photosystem II, has been demonstrated to be stable in quinoa, despite water stress (Morales et al., 2017). Amongst the treatments and time points in the three quinoa cultivars used in this study, the Fv/Fm ratio was maintained higher than 0.7, which is considered an optimal value (**Figures 4A–C**).

We observed that N stimulates a higher quantum yield of PSII (ϕPSII, which reflects the real quantum efficiency of PSII) and decreases the thermal dissipation (measured as NPQ). In contrast, as senescence progressed, the drought treatment was differentially affected with respect to ϕPSII and NPQ (**Figure 4** and **Table 1**). During senescence progress, ϕPSII was reduced to similar values independent of water treatment; however, NPQ was dependent on W and N interaction and G × W × T. The drought treatment increased NPQ in Faro under both HNS and LNS treatments, whereas UdeC9 was only induced under HN treatment and BO78 showed no significant difference within W or N treatment (**Figure 4**). This suggests that NPQ induction was not regulated equally by the same factors (water nitrogen and senescence progress) among genotypes.

We found that the enhancement of NPQ was negatively and closely related with *P_n_* reductions during senescence (**Figure 5**), suggesting a photoprotective role in dissipating the excess of excitation energy absorbed by PSII as heat. However, this effect was only found in Faro and UdeC9, but not in BO78. The lack of NPQ response in BO78 may imply that excess energy by NPQ is not the principal pathway to reduce ROS formation. Unlike Faro and UdeC9, which significantly reduced the chlorophyll content through the senescence progress, BO78 (the southern genotype) maintained their low Chl levels but accumulated betacyanin. The larger store of betacyanins in BO78 with respect to Faro and UdeC9 was almost dependent on N, W, and T interactions and was highest under HNS conditions (**Figure 6, Supplementary Table S2**, and **Table 2**). Betacyanins are water-soluble nitrogen-containing pigments with chemotaxonomical value (Brockington et al., 2011). Despite having comparable functions with anthocyanin, these pigments have never been found with anthocyanins in the same species (Brockington et al., 2011). These chromophores are antioxidant, and function by masking photosynthetic pigments and quenching free radicals (Gandia-Herrero and Garcia-Carmona, 2013). Water deficiency leads to accumulation of betacyanins in beet and *Amaranthus hypochondriacus* with concomitant enhancement of the antioxidant level (Casique-Arroyo et al., 2014). However, the accumulation of betacyanins in BO78 cannot be attributed to an increased antioxidant role in this quinoa genotype as there is increased lipid peroxidation (**Figure 3I**) and decrease of Fv/Fm and PSII activity (**Figure 4F**).

Nonetheless, betacyanins could also play an intermediary role in the conversion of cellular nitrogen compounds (Gins et al., 2002). Therefore, we suggest that accumulation of betacyanins in BO78 could be a mechanism to store N under conditions of protein degradation, such as water stress, to avoiding the production of toxic N-related compounds. Further analyses are necessary to determine the role of these molecules under drought and senescence conditions in red genotypes of quinoa.

### N and Terminal Drought Interaction Determine the Pigment Level Related to Thermal Dissipation in Quinoa

NPQ mechanisms are known to be dependent on the xanthophyll cycle (Demmig-Adams, 1990; Demmig-Adams and Adams, 1992). Both NPQ and many analyzed pigments (including chlorophylls, carotenoids, and betacyanins) were dependent on N × W interactions (**Table 2**). The progression of senescence influenced the level of the de-epoxidation state of xanthophylls (DEPS) through increased zeaxanthin and antheraxanthin, while the total size of the xanthophyll pool remained unchanged (**Supplementary Table S2**). These results were consistent with those reported for other woody species (García-Plazaola et al., 2003). In Faro, the carotenoid pigments were converted from zeaxanthin to violaxanthin. However, in UdeC9 and BO78, other pigments may be involved in the zeaxanthin increase such as lutein and β-carotene that were reduced on these genotypes (**Figure 6**).

The role of zeaxanthin in drought tolerance in plants has been described (Demmig-Adams et al., 1988; Zhao et al., 2014). Transgenic tobacco lines with zeaxanthin enhancement increased the drought tolerance of the plants (Zhao et al., 2014). In addition, lutein has been reported to be critical for efficient chlorophyll triplet quenching (Dall’Osto et al., 2006). We suggest that the higher capacity of xanthophyll interconversion to zeaxanthin in Faro, as well as lutein maintenance, was related to its heightened photoprotection through NPQ even under LNS conditions in contrast to UdeC9 and BO78.

When examining the DEPS versus NPQ relationship, it was only significant for plants developed at HN but not at LN (evaluated at *p* < 0.05) (**Figure 7**). The less pronounced increase of NPQ as DEPS increased suggests restricted plasticity of NPQ despite great changes in DEPS under LN conditions (**Figure 7**). We suggest that at LN supply the stoichiometry of PSII protein/pigment components could shift to maintain NPQ at the maximum level, but reduce the plant’s ability to further respond to environmental or/and intrinsic changes. It is well known that NPQ comprises at least two components. The fast relaxing component NPQf, related with zeaxanthin synthesis in the xanthophyll cycle and protonation PsbS protein (from the PSII harvesting complex), and the slowly reversible component NPQs, related with dark retention of zeaxanthin and photoinhibitory damage (Maxwell and Johnson, 2000; Bascuñán-Godoy et al., 2012). Our results suggest that under LN conditions the NPQs fraction would explain a higher proportion of NPQ, resulting in a reduced capacity for enhancement of dynamic heat dissipation (NPQf). Another interpretation could be related to a reduced proportion of N allocation to proteins involved in energy dissipation, such as PsbS, which is essential for inducing rapid formation of light-inducible thermal dissipation (Müller et al., 2001). Further work regarding the adjustment of the light harvesting reaction center complex and photosynthetic proteins will be necessary to understand the molecular basis of the complexities for thermal dissipation versus photoassimilation under LN stress conditions.

In summary, our physiological results suggest that N supply regulates the photosynthetic attributes and photochemical processes. The magnitude of thermal dissipation induction during senescence was dependent on both N and water availability. This was supported by the great interaction of N × W on xanthophyll pigments. The effect of N in the attributes related to photosynthesis and photoprotecction during senescence were often dependent on genotypes. Specifically, we suggest: (i) Faro which presented with differential root growth under LN conditions showed attributes that allow a greater tolerance to N and water deficit in comparison with UdeC9 and BO78 genotypes, (ii) the photoprotective induction of NPQ during senescence and drought-induced senescence is used by green genotypes, but not BO78, which accumulated betacyanins, and (iii) LN supply compromised the plasticity of the photoprotective attributes restricting further changes during drought-induced senescence.

## Conclusion

We conclude that N supply regulates photoprotection of the photosynthetic apparatus during senescence and drought-induced senescence progress. The photosynthetic capacity during senescence was independent of the terminal drought effect. NPQ related with the photoprotection of the photosynthetic apparatus through heat dissipation was induced during drought-induced senescence compared with control plants. The capacity of thermal dissipation during drought-induced senescence was regulated by N and water supply.

Our findings are important as exploration of different quinoa germplasm and identification of valuable traits could be used for screening and selecting nitrogen and water-stress tolerant genotypes. Better understanding of quinoa genotypes would enable crop expansion into poorer soils with limited irrigation.

## Author Contributions

LB-G designed the assays and led the writing of the manuscript. RÁ and CS grew both *C. quinoa* genotypes. KP, MG-T, and LC performed the assays and measurements. LB provided pigments assays and partook in discussions. CH conducted the statistical analysis. LB-G and LB led the supported projects and edited the manuscript. All authors read, edited and approved the final version of the manuscript for publication.

## Conflict of Interest Statement

The authors declare that the research was conducted in the absence of any commercial or financial relationships that could be construed as a potential conflict of interest.

## Footnotes

1 www.statsoft.com

## Supplementary Material

FIGURE S1 Plant phenotype and biomass of three genotypes of *Chenopodium quinoa* growing under different N regimens. **(A)** Four-month-old Faro (left), UdeC9 (middle), and BO78 (right) were grown at High Nitrogen (HN) and Low Nitrogen (LN) supplies. **(B)** Aerial: root biomass. Top part of the bars indicating aerial dry weight: black and grey correspond to HN and LN, respectively. Bottom part of the bars indicating roots biomass: white with stripes or white, correspond to HN or LN, respectively. Bars show mean values ± SE (*n* = 4). Photographs and samples were taken 2 weeks after flowering (14 DAF). Different letters represent significant differences at *p* < 0.05 using two-way ANOVA using genotypes (Faro, UdeC9, BO78) and N regimen (HN and LN) as independent factors.

TABLE S1 Statistical effect of Genotype, Nitrogen supply, water treatment and their interactions on yield (using three-way ANOVA analysis) and the effect of genotype, nitrogen supply, water treatment and time and their interactions on physiological attributes (Net photosynthetic rate (*P_n_*), stomatal conductance (*g_s_*), intrinsic water-use efficiency (_i_WUE), relative water content of leaves, proteins, lipid peroxidation (estimated by MDA), the maximal efficiency of PSII (Fv/Fm), quantum yield of PSII (ΦPSII) and the non-photochemical quenching (NPQ) using four-way ANOVA analysis.

TABLE S2 Effect of water stress on chlorophylls, xanthophylls, de-epoxidation state of xanthophyll cycle (DEPS), carotenoids, and betacyanins in three genotypes of *Chenopodium quinoa* grown at two levels of nitrogen supplementation. Fully expanded third leaves (from the top part of the plant) were used for measurements. Samples were taken at mid-morning at both 0 and 15 days of drought (14 and 29 DAF, respectively). Different letters represent significant differences between genotypes, treatments, and day of drought treatment *P* < 0.05 using four-way ANOVA. Newman–Keuls analysis (level of significance *P* < 0.05) was used as a post hoc test. Values are expressed in μm g^-1^ DW. Values are mean ± SE (*n* = 4).

TABLE S3 Effect of Genotype, Nitrogen supply, water treatment, time and their interactions effects on pigments using four- way ANOVA analysis.

## References

[B1] Alandia G. Jacobsen S.-E. Kyvsgaard N. C. Condori B. Liu F. (2016). Nitrogen sustains seed yield of quinoa under intermediate drought. *J. Agron. Crop Sci.* 202 281–291. 10.1111/jac.12155

[B2] Balazadeh S. Schildhauer J. Araújo W. L. Munné-Bosch S. Fernie A. R. Proost S. (2014). Reversal of senescence by N resupply to N-starved *Arabidopsis thaliana*: transcriptomic and metabolomic consequences. *J. Exp. Bot.* 65 3975–3992. 10.1093/jxb/eru119 24692653 PMC4106441

[B3] Bascuñán-Godoy L. Sanhueza C. Cuba M. Zuñiga G. E. Corcuera L. J. Bravo L. A. (2012). Cold-acclimation limits low temperature induced photoinhibition by promoting a higher photochemical quantum yield and a more effective PSII restoration in darkness in the Antarctic rather than the Andean ecotype of *Colobanthus quitensis* Kunt Bartl (Cariophyllaceae). *BMC Plant Biol.* 12:114. 10.1186/1471-2229-12-114 22827966 PMC3490872

[B4] Bascuñán-Godoy L. Alcaino C. Carvajal D. E. Sanhueza C. Montecinos S. Maldonado A. (2015). Ecophysiological responses to drought followed by re-watering of two native Chilean swamp forest plants: *Myrceugenia exsucca* (DC.) O. Berg and *Luma chequen* (Molina) A. Gray. *Gayana Bot.* 72 203–212. 10.4067/S0717-66432015000200004

[B5] Bascuñán-Godoy L. Reguera M. Abdel-Tawab Y. M. Blumwald E. (2016). Water deficit stress-induced changes in carbon and nitrogen partitioning in *Chenopodium quinoa* Willd. *Planta* 243 591–603. 10.1007/s00425-015-2424-z 26560134

[B6] Bendevis M. A. Sun Y. Rosenqvist E. Shabala S. Liu F. Jacobsen S.-E. (2014). Photoperiodic effects on short-pulse C-14 assimilation and overall carbon and nitrogen allocation patterns in contrasting quinoa cultivars. *Environ. Exp. Bot.* 104 9–15. 10.1016/j.envexpbot.2014.03.002

[B7] Berti M. Wilckens R. Hevia F. Serri H. Vidal I. Mendez C. (2000). Nitrogen fertilization in quinoa (*Chenopodium quinoa* Willd). Fertilizacion nitrogenada en quinoa (*Chenopodium quinoa* Willd). *Cien. Investig. Agrar.* 27 81–90. 10.7764/rcia.v27i2.999

[B8] Bradford M. M. (1976). A rapid and sensitive method for the quantitation of microgram quantities of protein utilizing the principle of protein-dye binding. *Anal. Biochem.* 7 248–254. 10.1016/0003-2697(76)90527-3 942051

[B9] Brockington S. F. Walker R. H. Glover B. J. Soltis P. S. Soltis D. E. (2011). Complex pigment evolution in the Caryophyllales. *New Phytol.* 190 854–864. 10.1111/j.1469-8137.2011.03687.x 21714182

[B10] Casique-Arroyo G. Martínez-Gallardo N. González de la Vara L. Delano-Frier J. P. (2014). Betacyanin biosynthetic genes and enzymes are differentially induced by (a)biotic stress in *Amaranthus hypochondriacus*. *PLoS One* 9:e99012. 10.1371/journal.pone.0099012 24896616 PMC4045864

[B11] Dall’Osto L. Lico C. Alric J. Giuliano G. Havaux M. Bassi R. (2006). Lutein is needed for efficient chlorophyll triplet quenching in the major LHCII antenna complex of higher plants and effective photoprotection *in vivo* under strong light. *BMC Plant Biol.* 6:32. 10.1186/1471-2229-6-32 17192177 PMC1769499

[B12] Demmig-Adams B. Winter K. Krüger A. Czygan F. C. (1988). Zeaxanthin and the heat dissipation of excess light energy in *Nerium oleander* exposed to a combination of high light and water stress. *Plant Physiol.* 87 17–24. 10.1104/pp.87.1.17 16666096 PMC1054692

[B13] Demmig-Adams B. (1990). Carotenoids and photoprotection in plants: a role for the xanthophyll zeaxanthin. *Biochim. Biophys. Acta* 1020 1–24. 10.1016/0005-2728(90)90088-L

[B14] Demmig-Adams B. Adams W. W. (1992). Photoprotection and other responses of plants to high light stress. *Annu. Rev. Plant Physiol. Plant Mol. Biol.* 43 599–626. 10.1146/annurev.pp.43.060192.003123

[B15] Egli D. B. Leggett J. E. Duncan W. G. (1976). Influence of N stress on leaf senescence and N redistribution in soybeans. *Agron. J.* 70 43–47. 10.2134/agronj1978.00021962007000010011x

[B16] FAO (2011) *Quinoa: An Ancient Crop to Contribute to World Food Security. Food and Agricultural Organization of the United Nations.* Available at: http://www.fao.org/docrep/017/aq287e/aq287e.pdf

[B17] Gaju O. Allard V. Martre P. Snape J. W. Heumez E. LeGouis J. (2011). Identification of traits to improve the nitrogen-use efficiency of wheat genotypes. *Field Crops Res.* 123 139–152. 10.1016/j.fcr.2011.05.010

[B18] Gan S. Amasino R. M. (1997). Making sense of senescence molecular genetic regulation and manipulation of leaf senescence. *Plant Physiol.* 113 313–319. 10.1104/pp.113.2.313 12223609 PMC158144

[B19] Gandia-Herrero F. Garcia-Carmona F. (2013). Biosynthesis of betalains: yellow and violet plant pigments. *Trends Plant Sci.* 18 334–343. 10.1016/j.tplants.2013.01.003 23395307

[B20] García-Plazaola J. I. Becerril J. M. (1999). A rapid high-performance liquid chromatography method to measure lipophilic antioxidants in stressed plants: simultaneous determination of carotenoids and tocopherols. *Phytochem. Anal.* 10 307–313. 10.1002/(SICI)1099-1565(199911/12)10:6<307::AID-PCA477>3.0.CO;2-L

[B21] García-Plazaola J. I. Becerril J. M. (2001). Seasonal changes in photosynthetic pigments and antioxidants in beech (*Fagus sylvatica*) in a mediterranean climate: implications for tree decline diagnosis. *Aust. J. Plant Physiol.* 28 225–232. 10.1071/PP00119

[B22] García-Plazaola J. I. Hernández A. Becerril J. M. (2003). Antioxidant and pigment composition during autumnal leaf senescence in woody deciduous species differing in their ecological traits. *Plant Biol.* 5 557–566. 10.1055/s-2003-44791

[B23] Gins M. S. Gins V. K. Kononkov P. F. (2002). Change in the biochemical composition of amaranth leaves during selection for increased amaranthine content. *Appl. Biochem. Microbiol.* 38 474–479. 10.1023/A:1019980821313 12391759

[B24] Gonzalez-Teuber M. Urzúa A. Plaza P. Bascunan-Godoy L. (2018). Effects of root endophytic fungi on response of *Chenopodium quinoa* to drought stress. *Plant Ecol.* 219 231–240. 10.1007/s11258-017-0791-1

[B25] Gregersen P. L. Culetic A. Boschian L. Krupinska K. (2013). Plant senescence and crop productivity. *Plant Mol. Biol.* 82 603–622. 10.1007/s11103-013-0013-8 23354836

[B26] Jacobsen S. E. (1997). Adaptation of quinoa (*Chenopodium quinoa*) to Northern European agriculture: studies on developmental pattern. *Euphytica* 96 41–48. 10.1023/A:1002992718009

[B27] Jacobsen S. E. Stølen O. (1993). Quinoa – morphology and phenology and prospects for its production as a new crop in Europe. *Eur. J. Agron.* 2 19–29. 10.1016/S1161-0301(14)80148-2

[B28] Kichey T. Hirel B. Heumez E. Dubois F. Le Gouis J. (2007). In winter wheat (*Triticum aestivum* L.), post-anthesis nitrogen uptake and remobilisation to the grain correlates with agronomic traits and nitrogen physiological markers. *Field Crops Res.* 102 22–32. 10.1016/j.fcr.2007.01.002

[B29] Kramer D. M. Johnson G. Kiirats O. Edwards G. E. (2004). New fluorescence parameters for the determination of QA redox state and excitation energy fluxes. *Photosynth. Res.* 79 209–218. 10.1023/B:PRES.0000015391.99477.0d 16228395

[B30] Lu C. Lu Q. Zhang J. Kuang T. (2001). Characterization of photosynthetic pigment composition, photosystem II photochemistry and thermal energy dissipation during leaf senescence of wheat plants grown in the field. *J. Exp. Bot.* 52 1805–1810. 10.1093/jexbot/52.362.1805 11520868

[B31] Lu Q. Wen X. Lu C. Zhang Q. Kuang T. (2003). Photoinhibition and photoprotection in senescent leaves of field-grown wheat plants. *Plant Physiol. Biochem.* 41 749–754. 10.1016/S0981-9428(03)00098-6

[B32] Lutz M. Bascunan-Godoy L. (2017). “The revival of Quinoa: a crop for health,” in *Superfood and Functional Food - An Overview and its Utilization to Processed Foods* ed. Shiomi N. (Rijeka: InTech).

[B33] Marschner P. (2012). *Marschner’s Mineral Nutrition of Higher Plants* 3rd Edn. London: Academic Press.

[B34] Maxclaux-Daubresse C. Quillere I. Gallais A. Hirel B. (2001). The challenge of remobilisation in plant nitrogen economy. A survey of physioagronomic and molecular approaches. *Ann. Appl. Biol.* 138 69–81. 10.1111/j.1744-7348.2001.tb00086.x

[B35] Masclaux-Daubresse C. Daniel-Vedele F. Dechorgnat J. Chardon F. Gaufichon L. Suzuki A. (2010). Nitrogen uptake, assimilation and remobilization in plants: challenges for sustainable and productive agriculture. *Ann. Bot.* 105 1141–1157. 10.1093/aob/mcq028 20299346 PMC2887065

[B36] Maxwell K. Johnson G. N. (2000). Chlorophyll fluorescence - a practical guide. *J. Exp. Bot.* 51 659–668. 10.1093/jexbot/51.345.659 10938857

[B37] Morales A. Zurita-Silva A. Maldonado J. Silva H. (2017). Transcriptional responses of chilean quinoa (*Chenopodium quinoa* Willd.) under water deficit conditions uncovers ABA-independent expression patterns. *Front. Plant Sci.* 8:216. 10.3389/fpls.2017.00216 28337209 PMC5340777

[B38] Müller P. Li X. P. Niyogi K. K. (2001). Non-photochemical quenching. A response to excess of light energy. *Plant Physiol.* 125 1558–1566. 10.1104/pp.125.4.1558 11299337 PMC1539381

[B39] Ortega-Villasante C. Rellán-Álvarez R. Del Campo F. F. Carpena-Ruiz R. O. Hernández L. E. (2005). Cellular damage induced by cadmium and mercury in *Medicago sativa*. *J. Exp. Bot.* 56 2239–2251. 10.1093/jxb/eri223 15996984

[B40] Pompelli M. F. Martins S. C. Antunes W. C. Chaves A. R. DaMatta F. M. (2010). Photosynthesis and photoprotection in coffee leaves is affected by nitrogen and light availabilities in winter conditions. *J. Plant Physiol.* 167 1052–1060. 10.1016/j.jplph.2010.03.001 20381192

[B41] Posch S. Warren C. R. Adams M. A. Guttenberger H. (2008). Photoprotective carotenoids and antioxidants are more affected by canopy position than by nitrogen supply in 21-year-old *Pinus radiata*. *Funct. Plant Biol.* 35 470–482. 10.1071/FP08124 32688804

[B42] Ramalho J. C. Campos P. S. Teixeira M. Nunes M. A. (1998). Nitrogen dependent changes in antioxidant systems and in fatty acid composition of chloroplast membranes from *Coffea arabica* L. plants submitted to high irradiance. *Plant Sci.* 135 115–124. 10.1016/S0168-9452(98)00073-9

[B43] Ramalho J. C. Pons T. L. Groeneveld H. W. Azinheira H. G. Nunes M. A. (2000). Photosynthetic acclimation to high light conditions in mature leaves of *Coffea arabica* L.: role of xanthophylls, quenching mechanisms and nitrogen nutrition. *Aust. J. Plant Physiol.* 27 43–51. 10.1071/PP99013

[B44] Ray D. K. Gerber J. S. MacDonald G. K. West P. C. (2015). Climate variation explains a third of global crop yield variability. *Nat. Commun.* 6:5989. 10.1038/ncomms6989 25609225 PMC4354156

[B45] Razzaghi F. Plauborg F. Jacobsen S. E. Jensen C. R. Andersen M. N. (2012). Effect of nitrogen and water availability of three soil types on yield, radiation use efficiency and evapotranspiration in field-grown quinoa. *Agric. Water Manag.* 109 20–29. 10.1016/j.agwat.2012.02.002

[B46] Reguera M. Peleg Z. Abdel-Tawab Y. M. Tumimbang E. B. Delatorre C. A. Blumwald E. (2013). Stress induced cytokinin synthesis increases drought tolerance through the coordinated regulation of carbon and nitrogen assimilation in rice. *Plant Physiol.* 163 1609–1622. 10.1104/pp.113.227702 24101772 PMC3850209

[B47] Ruiz-Carrasco K. Antognoni F. Coulibaly A. K. Lizardi S. Covarrubias A. Martínez E. A. (2011). Variation in salinity tolerance of four lowland genotypes of quinoa (*Chenopodium quinoa* Willd.) as assessed by growth, physiological traits, and sodium transporter gene expression. *Plant Physiol. Biochem.* 49 1333–1341. 10.1016/j.plaphy.2011.08.005 22000057

[B48] Sáez P. L. Bravo L. A. Latsague M. I. Toneatti M. J. Sánchez-Olate M. Ríos D. G. (2013). Light energy management in micropropagated plants of *Castanea sativa*, effects of photoinhibition. *Plant Sci.* 201 12–24. 10.1016/j.plantsci.2012.11.008 23352399

[B49] Thanapornpoonpong S. N. Vearasilp S. Pawelzik E. Gorinstein S. (2008). Influence of various nitrogen applications on protein and amino acid profiles of amaranth and quinoa. *J. Agric. Food Chem.* 56 11464–11470. 10.1021/jf802673x 19006392

[B50] Verhoeven A. S. Demmig-Adams B. Adams W. W. III. (1997). Enhanced employment of the xanthophyll cycle and thermal energy dissipation in spinach exposed to high light and N stress. *Plant Physiol.* 113 817–824. 10.1104/pp.113.3.817 12223645 PMC158201

[B51] Wang Y. Loake G. J. Chu C. (2013). Cross-talk of nitric oxide and reactive oxygen species in plant programed cell death. *Front. Plant Sci.* 4:314. 10.3389/fpls.2013.00314 23967004 PMC3744911

[B52] Yoo S. D. Greer D. H. Laing W. A. McManus M. T. (2003). Changes in photosynthetic efficiency and carotenoid composition in leaves of white clover at different developmental stages. *Plant Physiol. Biochem.* 41 887–893. 10.1016/S0981-9428(03)00138-4

[B53] Zhao Q. Wang G. Ji J. Jin C. Wu W. Zhao J. (2014). Over-expression of *Arabidopsis thaliana* β-carotene hydroxylase (chyB) gene enhances drought tolerance in transgenic tobacco. *J. Plant Biochem. Biotechnol.* 23 190–198. 10.1007/s13562-013-0201-2

