# Supplementary figures and images for: Nitrogen Supply Affects Photosynthesis and Photoprotective Attributes During Drought-Induced Senescence in Quinoa
Source: Front Plant Sci. 2018 Jul 30;9:994. doi: 10.3389/fpls.2018.00994 (PMC6077362; doi:10.3389/fpls.2018.00994)

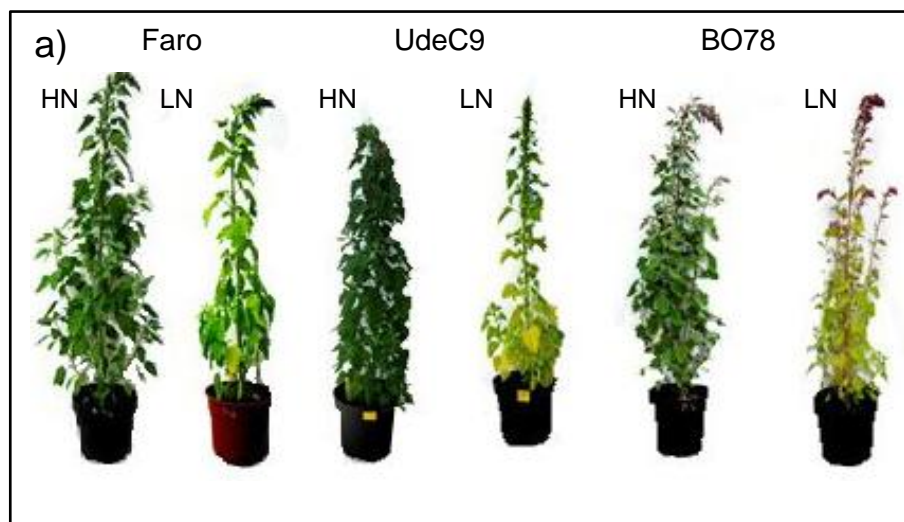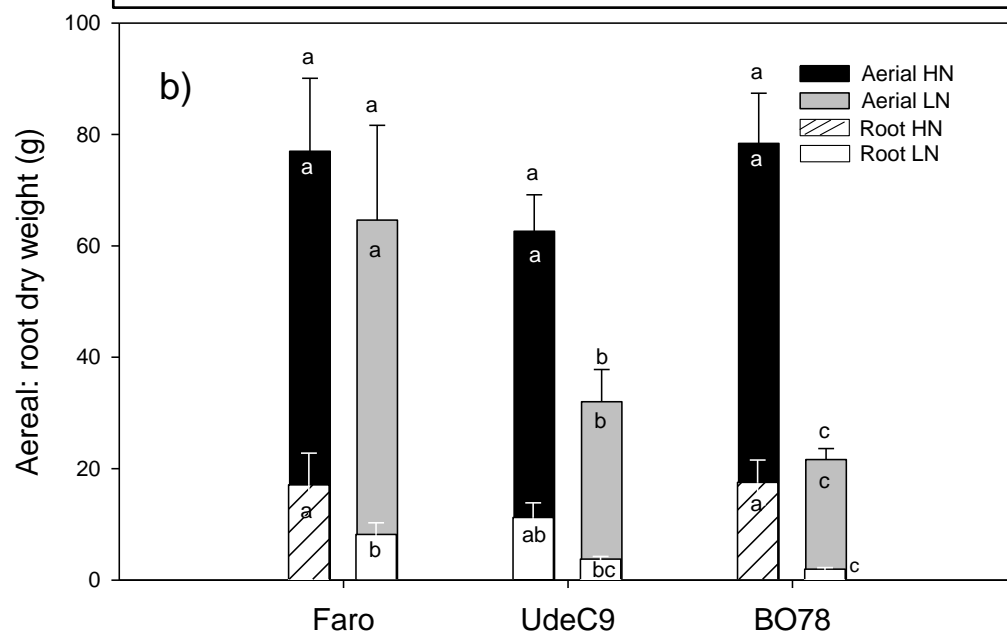

Supplement: FIGURE S1 — Plant phenotype and biomass of three genotypes of Chenopodium quinoa growing under different N regimens. (A) Four-month-old Faro (left), UdeC9 (middle), and BO78 (right) were grown at High Nitrogen (HN) and Low Nitrogen (LN) supplies. (B) Aerial: root biomass. Top part of the bars indicating aerial dry weight: black and grey correspond to HN and LN, respectively. Bottom part of the bars indicating roots biomass: white with stripes or white, correspond to HN or LN, respectively. Bars show mean values ± SE (n = 4). Photographs and samples were taken 2 weeks after flowering (14 DAF). Different letters represent significant differences at p < 0.05 using two-way ANOVA using genotypes (Faro, UdeC9, BO78) and N regimen (HN and LN) as independent factors. [file Image_1.PDF]
