# Supplementary material for: Nitrogen Supply Affects Photosynthesis and Photoprotective Attributes During Drought-Induced Senescence in Quinoa
Source: Front Plant Sci. 2018 Jul 30;9:994. doi: 10.3389/fpls.2018.00994 (PMC6077362; doi:10.3389/fpls.2018.00994)
Supplement: TABLE S1 — Statistical effect of Genotype, Nitrogen supply, water treatment and their interactions on yield (using three-way ANOVA analysis) and the effect of genotype, nitrogen supply, water treatment and time and their interactions on physiological attributes (Net photosynthetic rate (Pn), stomatal conductance (gs), intrinsic water-use efficiency (iWUE), relative water content of leaves, proteins, lipid peroxidation (estimated by MDA), the maximal efficiency of PSII (Fv/Fm), quantum yield of PSII (ΦPSII) and the non-photochemical quenching (NPQ) using four-way ANOVA analysis. [file Table_1.DOCX]

**Supplementary Table S1.** Statistical effect of Genotype, Nitrogen supply, water treatment and interactions on yield (using three- way ANOVA analysis) and the effect of Genotype, Nitrogen supply, water treatment and time and their interaction on physiological attributes (Net photosynthetic rate (*P_n_*), stomatal conductance (*g_s_*), intrinsic water-use efficiency (iWUE), relative water content of leaves (RWC), proteins, lipid peroxidation (estimated by MDA), the maximal efficiency of PSII (Fv/Fm), quantum yield of PSII (ΦPSII) and the non-photochemical quenching (NPQ)) using four- way ANOVA analysis.

| Yield | SS | Degr Freedom | MS | F | p |
| --- | --- | --- | --- | --- | --- |
|  | 26259,04 | 1 | 26259,04 | 857,9042 | 0,000000 |
| G | 788,78 | 2 | 394,39 | 12,8850 | 0,000064 |
| N | 523,45 | 1 | 523,45 | 17,1015 | 0,000211 |
| W | 631,87 | 1 | 631,87 | 20,6437 | 0,000063 |
| G*N | 59,85 | 2 | 29,93 | 0,9777 | 0,386199 |
| G*W | 263,15 | 2 | 131,57 | 4,2987 | 0,021412 |
| N*W | 72,19 | 1 | 72,19 | 2,3585 | 0,133597 |
| G*N*W | 55,27 | 2 | 27,63 | 0,9028 | 0,414666 |
| Error | 1071,29 | 35 | 30,61 |  |  |

| Photosynthesis | SS | Tegr FreeTom | MS | F | p |  |
| --- | --- | --- | --- | --- | --- | --- |
| Intercept | 16598,48 | 1 | 16598,48 | 904,1764 | 0,000000 |  |
| G | 691,03 | 2 | 345,52 | 18,8215 | 0,000000 |  |
| N | 153,34 | 1 | 153,34 | 8,3528 | 0,005185 |  |
| T | 942,96 | 1 | 942,96 | 51,3663 | 0,000000 |  |
| W | 49,00 | 1 | 49,00 | 2,6690 | 0,107014 |  |
| G*N | 123,07 | 2 | 61,53 | 3,3520 | 0,040979 |  |
| G*T | 10,48 | 2 | 5,24 | 0,2854 | 0,752600 |  |
| N*T | 198,54 | 1 | 198,54 | 10,8152 | 0,001606 |  |
| G*W | 25,50 | 2 | 12,75 | 0,6945 | 0,502872 |  |
| N*W | 11,84 | 1 | 11,84 | 0,6452 | 0,424684 |  |
| T*W | 49,00 | 1 | 49,00 | 2,6690 | 0,107014 |  |
| G*N*T | 5,57 | 2 | 2,79 | 0,1517 | 0,859527 |  |
| G*N*W | 15,36 | 2 | 7,68 | 0,4184 | 0,659775 |  |
| G*T*W | 25,50 | 2 | 12,75 | 0,6945 | 0,502872 |  |
| N*T*W | 11,84 | 1 | 11,84 | 0,6452 | 0,424684 |  |
| G*N*T*W | 15,36 | 2 | 7,68 | 0,4184 | 0,659775 |  |
| Error | 1229,96 | 67 | 18,36 |  |  |  |
|  |  |  |  |  |  |  |
| Conductance (gs) | SS | Tegr FreeTom | MS | F | p |  |
| Intercept | 2,745370 | 1 | 2,745370 | 558,0707 | 0,000000 |  |
| G | 0,033895 | 2 | 0,016948 | 3,4450 | 0,037659 |  |
| N | 0,010963 | 1 | 0,010963 | 2,2284 | 0,140184 |  |
| W | 0,050119 | 1 | 0,050119 | 10,1880 | 0,002153 |  |
| T | 0,141819 | 1 | 0,141819 | 28,8286 | 0,000001 |  |
| G*N | 0,015055 | 2 | 0,007527 | 1,5301 | 0,223979 |  |
| G*W | 0,001680 | 2 | 0,000840 | 0,1707 | 0,843435 |  |
| N*W | 0,005813 | 1 | 0,005813 | 1,1816 | 0,280930 |  |
| G*T | 0,003971 | 2 | 0,001986 | 0,4036 | 0,669510 |  |
| N*T | 0,045054 | 1 | 0,045054 | 9,1584 | 0,003510 |  |
| W*T | 0,050119 | 1 | 0,050119 | 10,1880 | 0,002153 |  |
| G*N*W | 0,002363 | 2 | 0,001181 | 0,2402 | 0,787174 |  |
| G*N*T | 0,042350 | 2 | 0,021175 | 4,3043 | 0,017432 |  |
| G*W*T | 0,001680 | 2 | 0,000840 | 0,1707 | 0,843435 |  |
| N*W*T | 0,005813 | 1 | 0,005813 | 1,1816 | 0,280930 |  |
| G*N*W*T | 0,002363 | 2 | 0,001181 | 0,2402 | 0,787174 |  |
| Error | 0,329599 | 67 | 0,004919 |  |  |  |
|  |  |  |  |  |  |  |
| WUE | SS | Tegr FreeTom | MS | F | p |  |
| Intercept | 659204,0 | 1 | 659204,0 | 1576,615 | 0,000000 |  |
| G | 5177,9 | 2 | 2588,9 | 6,192 | 0,003408 |  |
| N | 4185,1 | 1 | 4185,1 | 10,010 | 0,002342 |  |
| T | 8139,1 | 1 | 8139,1 | 19,466 | 0,000038 |  |
| W | 248,1 | 1 | 248,1 | 0,593 | 0,443839 |  |
| G*N | 50,0 | 2 | 25,0 | 0,060 | 0,941969 |  |
| G*T | 319,7 | 2 | 159,9 | 0,382 | 0,683758 |  |
| N*T | 2898,4 | 1 | 2898,4 | 6,932 | 0,010501 |  |
| G*W | 458,0 | 2 | 229,0 | 0,548 | 0,580873 |  |
| N*W | 1746,1 | 1 | 1746,1 | 4,176 | 0,044931 |  |
| T*W | 8139,1 | 1 | 8139,1 | 19,466 | 0,000038 |  |
| G*N*T | 77,5 | 2 | 38,8 | 0,093 | 0,911562 |  |
| G*N*W | 3064,9 | 2 | 1532,4 | 3,665 | 0,030864 |  |
| G*T*W | 319,7 | 2 | 159,9 | 0,382 | 0,683758 |  |
| N*T*W | 2898,4 | 1 | 2898,4 | 6,932 | 0,010501 |  |
| G*N*T*W | 77,5 | 2 | 38,8 | 0,093 | 0,911562 |  |
| Error | 28013,6 | 67 | 418,1 |  |  |  |
|  |  |  |  |  |  |  |
| RWC | SS | Tegr FreeTom | MS | F | p |  |
| Intercept | 933996,3 | 1 | 933996,3 | 17493,87 | 0,000000 |  |
| G | 1069,5 | 2 | 534,7 | 10,02 | 0,000095 |  |
| W | 1574,3 | 1 | 1574,3 | 29,49 | 0,000000 |  |
| N | 243,1 | 1 | 243,1 | 4,55 | 0,034883 |  |
| T | 1014,6 | 1 | 1014,6 | 19,00 | 0,000028 |  |
| G*W | 368,3 | 2 | 184,2 | 3,45 | 0,034950 |  |
| G*N | 555,0 | 2 | 277,5 | 5,20 | 0,006843 |  |
| W*N | 5,1 | 1 | 5,1 | 0,10 | 0,757505 |  |
| G*T | 82,3 | 2 | 41,1 | 0,77 | 0,465027 |  |
| W*T | 1017,1 | 1 | 1017,1 | 19,05 | 0,000027 |  |
| N*T | 13,5 | 1 | 13,5 | 0,25 | 0,616211 |  |
| G*W*N | 88,4 | 2 | 44,2 | 0,83 | 0,439311 |  |
| G*W*T | 799,5 | 2 | 399,7 | 7,49 | 0,000863 |  |
| G*N*T | 57,7 | 2 | 28,9 | 0,54 | 0,583901 |  |
| W*N*T | 100,7 | 1 | 100,7 | 1,89 | 0,172241 |  |
| G*W*N*T | 228,4 | 2 | 114,2 | 2,14 | 0,122257 |  |
| Error | 6406,8 | 120 | 53,4 |  |  |  |
|  |  |  |  |  |  |  |
| Proteins | SS | Tegr FreeTom | MS | F | p |  |
| Intercept | 5182902 | 1 | 5182902 | 1054,140 | 0,000000 |  |
| G | 105192 | 2 | 52596 | 10,697 | 0,000088 |  |
| N | 99765 | 1 | 99765 | 20,291 | 0,000026 |  |
| W | 7616 | 1 | 7616 | 1,549 | 0,217428 |  |
| T | 81438 | 1 | 81438 | 16,564 | 0,000122 |  |
| G*N | 37455 | 2 | 18727 | 3,809 | 0,026901 |  |
| G*W | 43792 | 2 | 21896 | 4,453 | 0,015115 |  |
| N*W | 397 | 1 | 397 | 0,081 | 0,777252 |  |
| G*T | 140126 | 2 | 70063 | 14,250 | 0,000006 |  |
| N*T | 42365 | 1 | 42365 | 8,617 | 0,004503 |  |
| W*T | 7616 | 1 | 7616 | 1,549 | 0,217428 |  |
| G*N*W | 1260 | 2 | 630 | 0,128 | 0,879961 |  |
| G*N*T | 14295 | 2 | 7148 | 1,454 | 0,240656 |  |
| G*W*T | 43792 | 2 | 21896 | 4,453 | 0,015115 |  |
| N*W*T | 397 | 1 | 397 | 0,081 | 0,777252 |  |
| G*N*W*T | 1260 | 2 | 630 | 0,128 | 0,879961 |  |
| Error | 344170 | 70 | 4917 |  |  |  |
|  |  |  |  |  |  |  |
| MDA | SS | Tegr FreeTom | MS | F | p |  |
| Intercept | 11592,48 | 1 | 11592,48 | 391,0640 | 0,000000 |  |
| G | 233,97 | 2 | 116,99 | 3,9465 | 0,024046 |  |
| N | 44,75 | 1 | 44,75 | 1,5097 | 0,223547 |  |
| W | 139,91 | 1 | 139,91 | 4,7198 | 0,033413 |  |
| T | 1010,49 | 1 | 1010,49 | 34,0881 | 0,000000 |  |
| G*N | 62,48 | 2 | 31,24 | 1,0539 | 0,354380 |  |
| G*W | 106,97 | 2 | 53,49 | 1,8043 | 0,172615 |  |
| N*W | 11,78 | 1 | 11,78 | 0,3973 | 0,530647 |  |
| G*T | 359,01 | 2 | 179,50 | 6,0554 | 0,003850 |  |
| N*T | 18,75 | 1 | 18,75 | 0,6326 | 0,429257 |  |
| W*T | 139,91 | 1 | 139,91 | 4,7198 | 0,033413 |  |
| G*N*W | 79,07 | 2 | 39,53 | 1,3336 | 0,270527 |  |
| G*N*T | 16,24 | 2 | 8,12 | 0,2739 | 0,761263 |  |
| G*W*T | 106,97 | 2 | 53,49 | 1,8043 | 0,172615 |  |
| N*W*T | 11,78 | 1 | 11,78 | 0,3973 | 0,530647 |  |
| G*N*W*T | 79,07 | 2 | 39,53 | 1,3336 | 0,270527 |  |
| Error | 1956,47 | 66 | 29,64 |  |  |  |
|  |  |  |  |  |  |  |
| Fv/Fm | SS | Tegr FreeTom | MS | F | p |  |
| Intercept | 58,93080 | 1 | 58,93080 | 80420,36 | 0,000000 |  |
| G | 0,00053 | 2 | 0,00027 | 0,36 | 0,696360 |  |
| N | 0,04819 | 1 | 0,04819 | 65,76 | 0,000000 |  |
| T | 0,00004 | 1 | 0,00004 | 0,05 | 0,822888 |  |
| W | 0,00881 | 1 | 0,00881 | 12,03 | 0,000888 |  |
| G*N | 0,00567 | 2 | 0,00284 | 3,87 | 0,025332 |  |
| G*T | 0,01138 | 2 | 0,00569 | 7,77 | 0,000882 |  |
| N*T | 0,00449 | 1 | 0,00449 | 6,13 | 0,015669 |  |
| G*W | 0,00153 | 2 | 0,00077 | 1,05 | 0,356948 |  |
| N*W | 0,00003 | 1 | 0,00003 | 0,05 | 0,830641 |  |
| T*W | 0,00004 | 1 | 0,00004 | 0,05 | 0,822888 |  |
| G*N*T | 0,00043 | 2 | 0,00021 | 0,29 | 0,748426 |  |
| G*N*W | 0,00185 | 2 | 0,00093 | 1,26 | 0,289092 |  |
| G*T*W | 0,01138 | 2 | 0,00569 | 7,77 | 0,000882 |  |
| N*T*W | 0,00449 | 1 | 0,00449 | 6,13 | 0,015669 |  |
| G*N*T*W | 0,00043 | 2 | 0,00021 | 0,29 | 0,748426 |  |
| Error | 0,05276 | 72 | 0,00073 |  |  |  |
|  |  |  |  |  |  |  |
| PSII | SS | Tegr FreeTom | MS | F | p |  |
| Intercept | 7,188630 | 1 | 7,188630 | 1597,626 | 0,000000 |  |
| G | 0,092730 | 2 | 0,046365 | 10,304 | 0,000116 |  |
| N | 0,437627 | 1 | 0,437627 | 97,260 | 0,000000 |  |
| W | 0,000756 | 1 | 0,000756 | 0,168 | 0,683098 |  |
| T | 0,498608 | 1 | 0,498608 | 110,812 | 0,000000 |  |
| G*N | 0,000262 | 2 | 0,000131 | 0,029 | 0,971294 |  |
| G*W | 0,000861 | 2 | 0,000431 | 0,096 | 0,908855 |  |
| N*W | 0,001331 | 1 | 0,001331 | 0,296 | 0,588181 |  |
| G*T | 0,030464 | 2 | 0,015232 | 3,385 | 0,039345 |  |
| N*T | 0,280630 | 1 | 0,280630 | 62,368 | 0,000000 |  |
| W*T | 0,000756 | 1 | 0,000756 | 0,168 | 0,683098 |  |
| G*N*W | 0,003336 | 2 | 0,001668 | 0,371 | 0,691589 |  |
| G*N*T | 0,019081 | 2 | 0,009540 | 2,120 | 0,127428 |  |
| G*W*T | 0,000861 | 2 | 0,000431 | 0,096 | 0,908855 |  |
| N*W*T | 0,001331 | 1 | 0,001331 | 0,296 | 0,588181 |  |
| G*N*W*T | 0,003336 | 2 | 0,001668 | 0,371 | 0,691589 |  |
| Error | 0,323969 | 72 | 0,004500 |  |  |  |
|  |  |  |  |  |  |  |
| NPQ | SS | Tegr FreeTom | MS | F | p |  |
| Intercept | 596,8671 | 1 | 596,8671 | 4698,143 | 0,000000 |  |
| G | 2,3454 | 2 | 1,1727 | 9,231 | 0,000270 |  |
| N | 9,4942 | 1 | 9,4942 | 74,732 | 0,000000 |  |
| T | 0,6060 | 1 | 0,6060 | 4,770 | 0,032215 |  |
| W | 5,8651 | 1 | 5,8651 | 46,166 | 0,000000 |  |
| G*N | 0,0377 | 2 | 0,0189 | 0,148 | 0,862277 |  |
| G*T | 0,8576 | 2 | 0,4288 | 3,375 | 0,039702 |  |
| N*T | 0,0012 | 1 | 0,0012 | 0,009 | 0,923212 |  |
| G*W | 2,6732 | 2 | 1,3366 | 10,521 | 0,000098 |  |
| N*W | 6,1726 | 1 | 6,1726 | 48,587 | 0,000000 |  |
| T*W | 0,6060 | 1 | 0,6060 | 4,770 | 0,032215 |  |
| G*N*T | 0,0774 | 2 | 0,0387 | 0,305 | 0,738211 |  |
| G*N*W | 0,4076 | 2 | 0,2038 | 1,604 | 0,208176 |  |
| G*T*W | 0,8576 | 2 | 0,4288 | 3,375 | 0,039702 |  |
| N*T*W | 0,0012 | 1 | 0,0012 | 0,009 | 0,923212 |  |
| G*N*T*W | 0,0774 | 2 | 0,0387 | 0,305 | 0,738211 |  |
| Error | 9,1471 | 72 | 0,1270 |  |  |  |
|  |  |  |  |  |  |  |
|  |  |  |  |  |  |  |
|  |  |  |  |  |  |  |
|  |  |  |  |  |  |  |
|  |  |  |  |  |  |  |
|  |  |  |  |  |  |  |
|  |  |  |  |  |  |  |
|  |  |  |  |  |  |  |
