# Supplementary material for: Nitrogen Supply Affects Photosynthesis and Photoprotective Attributes During Drought-Induced Senescence in Quinoa
Source: Front Plant Sci. 2018 Jul 30;9:994. doi: 10.3389/fpls.2018.00994 (PMC6077362; doi:10.3389/fpls.2018.00994)
Supplement: TABLE S2 — Effect of water stress on chlorophylls, xanthophylls, de-epoxidation state of xanthophyll cycle (DEPS), carotenoids, and betacyanins in three genotypes of Chenopodium quinoa grown at two levels of nitrogen supplementation. Fully expanded third leaves (from the top part of the plant) were used for measurements. Samples were taken at mid-morning at both 0 and 15 days of drought (14 and 29 DAF, respectively). Different letters represent significant differences between genotypes, treatments, and day of drought treatment P < 0.05 using four-way ANOVA. Newman–Keuls analysis (level of significance P < 0.05) was used as a post hoc test. Values are expressed in μm g-1 DW. Values are mean ± SE (n = 4). [file Table_2.DOCX]

**Supplemental Table S2.** Effect of water stress on chlorophylls, xanthophylls, de-epoxidation state of xanthophyll cycle (DEPS), carotenoids, and betacyanins in three genotypes of *Chenopodium quinoa* grown at two levels of nitrogen supplementation. Fully expanded third leaves (from the top part of the plant) were used for measurements. Samples were taken at mid-morning at both 0 and 15 days of drought (14 and 29 DAF, respectively). Different letters represent significant differences between genotypes, treatments, and day of drought treatment P<0.05 using four-way ANOVA. Newman–Keuls analysis (level of significance P<0.05) was used as a post hoc test. Values are expressed in µm g^-1^ DW. Values are mean ± SE (n=4).

| G | N | W | Day | Chl *a* | Chl *b* | Vio | Ant | Zea | V+A+Z | DEPS | Neo | Lut | B-car | Betacyanins |
| --- | --- | --- | --- | --- | --- | --- | --- | --- | --- | --- | --- | --- | --- | --- |
|  | HN | C | 0 | 3.68±0.90(a) | 0.98±0.25(a) | 0.41±0.10(ab) | 0.02±0.01(e) | 0.029±0.009(cd) | 0.46±0.11(a) | 0.09±0.01(ij) | 0.29±0.07(a) | 0.78±0.18(a) | 0.41±0.10(a) | 1,5±0,4(fg) |
|  | LN | C | 0 | 2.16±0.49(bc) | 0.54±0.14(ab) | 0.27±0.05(bc) | 0.03±0.00(de) | 0.023±0.003(cd) | 0.32±0.05(abc) | 0.12±0.02(hij) | 0.16±0.04(bc) | 0.48±0.12(bcd) | 0.24±0.06(bc) | 0,9±0,2(gh) |
| Faro | HN | C | 15 | 1.87±0.26(cd) | 0.44±0.08(bcd) | 0.26±0.03(bc) | 0.04±0.01(bcde) | 0.038±0.001(cd) | 0.34±0.02(ab) | 0.18±0.03(ghi) | 0.12±0.03(cde) | 0.39±0.05(bcd) | 0.17±0.04(cde) | 1,1±0,1(g) |
|  | LN | C | 15 | 1.46±0.39(cdf) | 0.32±0.08(cde) | 0.17±0.01(cde) | 0.04±0.01(bcde) | 0.033±0.004(b) | 0.25±0.03(bc) | 0.21±0.01(fgh) | 0.07±0.00(cdef) | 0.22±0.02(cde) | 0.10±0.01(cde) | 1,0±0,1(g) |
|  | HN | S | 15 | 1.50±0.46(cdf) | 0.38±0.12(cdf) | 0.18±0.02(cde) | 0.06±0.01(ab) | 0.108±0.021(cd) | 0.35±0.01(ab) | 0.40±0.06(bc) | 0.14±0.02(bcd) | 0.41±0.06(bcd) | 0.22±0.02(cd) | 1,8±0,8(efgh) |
|  | LN | S | 15 | 0.75±0.02(defg) | 0.17±0.00(def) | 0.09±0.03(de) | 0.07±0.02(a) | 0.173±0.047(a) | 0.34±0.10(ab) | 0.63±0.02(a) | 0.10±0.02(cdef) | 0.28±0.06(de) | 0.16±0.04(cde) | 2,4±0,3(e) |
|  |  |  |  |  |  |  |  |  |  |  |  |  |  |  |
|  | HN | C | 0 | 3.27±0.94(a) | 0.75±0.21(ab) | 0.43±0.13(a) | 0.03±0.00(de) | 0.014±0.007(d) | 0.48±0.14(a) | 0.06±0.02(j) | 0.24±0.07(ab) | 0.61±0.15(ab) | 0.39±0.13(ab) | 0,7±0,1(h) |
|  | LN | C | 0 | 0.46±0.15(efg) | 0.08±0.03(ef) | 0.14±0.03(cde) | 0.06±0.01(abc) | 0.050±0.004(cd) | 0.25±0.04(bc) | 0.33±0.02(cde) | 0.04±0.01(ef) | 0.17±0.03(de) | 0.07±0.02(de) | 0,9±0,1 (g) |
| UdeC9 | HN | C | 15 | 0.86±0.29(cdefg) | 0.20±0.07(def) | 0.15±0.02(cde) | 0.04±0.01(bcde) | 0.035±0.007(cd) | 0.22±0.01(bc) | 0.24±0.06(efg) | 0.07±0.02(cdef) | 0.23±0.06(cde) | 0.11±0.03(cde) | 0,9±0,1(g) |
|  | LN | C | 15 | 0.98±0.38(cdefg) | 0.20±0.09(def) | 0.12±0.02(cde) | 0.05±0.02(abcd) | 0.053±0.012(c) | 0.23±0.05(bc) | 0.35±0.02(bcd) | 0.06±0.02(def) | 0.23±0.06(cde) | 0.13±0.04(cde) | 1,5±0,3(f) |
|  | HN | S | 15 | 0.64±0.22(cdefg) | 0.13±0.05(def) | 0.15±0.03(cde) | 0.04±0.01(bcde) | 0.033±0.005(cd) | 0.22±0.04(bc) | 0.24±0.03(efg) | 0.05±0.02(def) | 0.20±0.04(de) | 0.09±0.02(cde) | 2,0±0,2(e) |
|  | LN | S | 15 | 0.11±0.07(g) | 0.02±0.01(f) | 0.07±0.01(e) | 0.05±0.01(abcde) | 0.051±0.009(c) | 0.17±0.03(bc) | 0.44±0.02(b) | 0.02±0.00(f) | 0.12±0.01(e) | 0.03±0.004(e) | 1,9±0,2(ef) |
|  |  |  |  |  |  |  |  |  |  |  |  |  |  |  |
|  | HN | C | 0 | 1.72±0.33(cde) | 0.41±0.09(ab) | 0.25±0.02(bcd) | 0.03±0.00(de) | 0.019±0.003(cd) | 0.30±0.03(abc) | 0.11±0.01(ij) | 0.14±0.03(bcd) | 0.39±0.07(bcd) | 0.22±0.04(cd) | 2,1±0,2(e) |
|  | LN | C | 0 | 1.03±0.35(cdfg) | 0.21±0.08(efg) | 0.21±0.07(cde) | 0.06±0.02(abc) | 0.047±0.017(cd) | 0.31±0.10(abc) | 0.24±0.00(efg) | 0.09±0.03(cdef) | 0.28±0.10(cde) | 0.12±0.04(cde) | 3,9±0,1(d) |
| BO78 | HN | C | 15 | 0.90±0.37(cdefg) | 0.22±0.10(cdef) | 0.14±0.04(cde) | 0.03±0.01(de) | 0.024±0.008(cd) | 0.19±0.03(bc) | 0.23±0.09(fg) | 0.08±0.03(cdef) | 0.27±0.08(cde) | 0.12±0.05(cde) | 9,8±0,3(c) |
|  | LN | C | 15 | 0.85±0.06(cdefg) | 0.17±0.01(cdef) | 0.11±0.02(cde) | 0.03±0.01(de) | 0.032±0.007(cd) | 0.17±0.04(bc) | 0.28±0.01(def | 0.06±0.003(cdef) | 0.18±0.01(de) | 0.11±0.01(cde) | 11,0±1,7(c) |
|  | HN | S | 15 | 0.23±0.09(g) | 0.05±0.02(def) | 0.07±0.01(e) | 0.04±0.00(cde) | 0.033±0.005(cd) | 0.14±0.01(c) | 0.38±0.06(bc) | 0.02±0.01(ef) | 0.13±0.02(e) | 0.04±0.01(e) | 23,9±1,2(a) |
|  | LN | S | 15 | 0.45±0.15(g) | 0.09±0.03(ef) | 0.10±0.01(e) | 0.05±0.00(abcde) | 0.050±0.003(c) | 0.20±0.01(bc) | 0.38±0.02(bc) | 0.04±0.01(ef) | 0.14±0.02(e) | 0.06±0.01(e) | 14,0±0,3(b) |
