# Supplementary material for: Nitrogen Supply Affects Photosynthesis and Photoprotective Attributes During Drought-Induced Senescence in Quinoa
Source: Front Plant Sci. 2018 Jul 30;9:994. doi: 10.3389/fpls.2018.00994 (PMC6077362; doi:10.3389/fpls.2018.00994)
Supplement: TABLE S3 — Effect of Genotype, Nitrogen supply, water treatment, time and their interactions effects on pigments using four- way ANOVA analysis. [file Table_3.DOCX]

**Supplementary Table S3.** Effect of Genotype, Nitrogen supply, water treatment and time and their interaction effects on pigments using four- way ANOVA analysis.

|  |  |  |  |  |  |  |
| --- | --- | --- | --- | --- | --- | --- |
|  |  |  |  |  |  |  |
| Chlorophyll a | SS | Tegr FreeTom | MS | F | p |  |
| Intercept | 155,1030 | 1 | 155,1030 | 234,8767 | 0,000000 |  |
| G | 17,9787 | 2 | 8,9894 | 13,6128 | 0,000021 |  |
| N | 18,4606 | 1 | 18,4606 | 27,9554 | 0,000003 |  |
| T | 0,0200 | 1 | 0,0200 | 0,0303 | 0,862483 |  |
| W | 24,6157 | 1 | 24,6157 | 37,2763 | 0,000000 |  |
| G*N | 5,6637 | 2 | 2,8318 | 4,2883 | 0,019342 |  |
| G*T | 0,9211 | 2 | 0,4606 | 0,6974 | 0,502837 |  |
| N*T | 0,0001 | 1 | 0,0001 | 0,0002 | 0,988183 |  |
| G*W | 1,7267 | 2 | 0,8634 | 1,3074 | 0,279981 |  |
| N*W | 7,9042 | 1 | 7,9042 | 11,9696 | 0,001144 |  |
| T*W | 0,0200 | 1 | 0,0200 | 0,0303 | 0,862483 |  |
| G*N*T | 0,2293 | 2 | 0,1147 | 0,1736 | 0,841137 |  |
| G*N*W | 1,9865 | 2 | 0,9933 | 1,5041 | 0,232499 |  |
| G*T*W | 0,9211 | 2 | 0,4606 | 0,6974 | 0,502837 |  |
| N*T*W | 0,0001 | 1 | 0,0001 | 0,0002 | 0,988183 |  |
| G*N*T*W | 0,2293 | 2 | 0,1147 | 0,1736 | 0,841137 |  |
| Error | 31,6972 | 48 | 0,6604 |  |  |  |
|  |  |  |  |  |  |  |
| Chlorophyll b | SS | Tegr FreeTom | MS | F | p |  |
| Intercept | 8,716549 | 1 | 8,716549 | 206,2459 | 0,000000 |  |
| G | 1,423212 | 2 | 0,711606 | 16,8376 | 0,000003 |  |
| N | 1,286278 | 1 | 1,286278 | 30,4352 | 0,000001 |  |
| T | 0,002805 | 1 | 0,002805 | 0,0664 | 0,797802 |  |
| W | 1,591648 | 1 | 1,591648 | 37,6606 | 0,000000 |  |
| G*N | 0,271493 | 2 | 0,135747 | 3,2120 | 0,049072 |  |
| G*T | 0,041468 | 2 | 0,020734 | 0,4906 | 0,615300 |  |
| N*T | 0,000354 | 1 | 0,000354 | 0,0084 | 0,927489 |  |
| G*W | 0,195004 | 2 | 0,097502 | 2,3070 | 0,110496 |  |
| N*W | 0,511950 | 1 | 0,511950 | 12,1135 | 0,001076 |  |
| T*W | 0,002805 | 1 | 0,002805 | 0,0664 | 0,797802 |  |
| G*N*T | 0,010493 | 2 | 0,005247 | 0,1241 | 0,883539 |  |
| G*N*W | 0,102802 | 2 | 0,051401 | 1,2162 | 0,305318 |  |
| G*T*W | 0,041468 | 2 | 0,020734 | 0,4906 | 0,615300 |  |
| N*T*W | 0,000354 | 1 | 0,000354 | 0,0084 | 0,927489 |  |
| G*N*T*W | 0,010493 | 2 | 0,005247 | 0,1241 | 0,883539 |  |
| Error | 2,028619 | 48 | 0,042263 |  |  |  |
|  |  |  |  |  |  |  |
| Violaxanthin | SS | Tegr FreeTom | MS | F | p |  |
| Intercept | 3,162791 | 1 | 3,162791 | 323,9148 | 0,000000 |  |
| G | 0,095746 | 2 | 0,047873 | 4,9029 | 0,011547 |  |
| N | 0,173925 | 1 | 0,173925 | 17,8123 | 0,000108 |  |
| T | 0,015135 | 1 | 0,015135 | 1,5501 | 0,219170 |  |
| W | 0,402101 | 1 | 0,402101 | 41,1809 | 0,000000 |  |
| G*N | 0,058274 | 2 | 0,029137 | 2,9840 | 0,060048 |  |
| G*T | 0,001453 | 2 | 0,000726 | 0,0744 | 0,928412 |  |
| N*T | 0,000066 | 1 | 0,000066 | 0,0067 | 0,934962 |  |
| G*W | 0,004668 | 2 | 0,002334 | 0,2390 | 0,788314 |  |
| N*W | 0,068185 | 1 | 0,068185 | 6,9831 | 0,011077 |  |
| T*W | 0,015135 | 1 | 0,015135 | 1,5501 | 0,219170 |  |
| G*N*T | 0,002250 | 2 | 0,001125 | 0,1152 | 0,891431 |  |
| G*N*W | 0,048778 | 2 | 0,024389 | 2,4978 | 0,092905 |  |
| G*T*W | 0,001453 | 2 | 0,000726 | 0,0744 | 0,928412 |  |
| N*T*W | 0,000066 | 1 | 0,000066 | 0,0067 | 0,934962 |  |
| G*N*T*W | 0,002250 | 2 | 0,001125 | 0,1152 | 0,891431 |  |
| Error | 0,468685 | 48 | 0,009764 |  |  |  |
|  |  |  |  |  |  |  |
| Anteraxanthin | SS | Tegr FreeTom | MS | F | p |  |
| Intercept | 0,125651 | 1 | 0,125651 | 441,8109 | 0,000000 |  |
| G | 0,000257 | 2 | 0,000128 | 0,4517 | 0,639244 |  |
| N | 0,002953 | 1 | 0,002953 | 10,3834 | 0,002287 |  |
| T | 0,001342 | 1 | 0,001342 | 4,7201 | 0,034781 |  |
| W | 0,000897 | 1 | 0,000897 | 3,1544 | 0,082063 |  |
| G*N | 0,000854 | 2 | 0,000427 | 1,5016 | 0,233044 |  |
| G*T | 0,000239 | 2 | 0,000120 | 0,4208 | 0,658926 |  |
| N*T | 0,000035 | 1 | 0,000035 | 0,1244 | 0,725813 |  |
| G*W | 0,004171 | 2 | 0,002085 | 7,3324 | 0,001664 |  |
| N*W | 0,001973 | 1 | 0,001973 | 6,9376 | 0,011326 |  |
| T*W | 0,001342 | 1 | 0,001342 | 4,7201 | 0,034781 |  |
| G*N*T | 0,000087 | 2 | 0,000043 | 0,1521 | 0,859314 |  |
| G*N*W | 0,001035 | 2 | 0,000517 | 1,8193 | 0,173143 |  |
| G*T*W | 0,000239 | 2 | 0,000120 | 0,4208 | 0,658926 |  |
| N*T*W | 0,000035 | 1 | 0,000035 | 0,1244 | 0,725813 |  |
| G*N*T*W | 0,000087 | 2 | 0,000043 | 0,1521 | 0,859314 |  |
| Error | 0,013651 | 48 | 0,000284 |  |  |  |
|  |  |  |  |  |  |  |
| Zeaxanthin | SS | Tegr FreeTom | MS | F | p |  |
| Intercept | 0,132177 | 1 | 0,132177 | 266,8761 | 0,000000 |  |
| G | 0,007271 | 2 | 0,003635 | 7,3399 | 0,001655 |  |
| N | 0,004976 | 1 | 0,004976 | 10,0467 | 0,002657 |  |
| T | 0,009311 | 1 | 0,009311 | 18,7996 | 0,000074 |  |
| W | 0,011268 | 1 | 0,011268 | 22,7514 | 0,000018 |  |
| G*N | 0,000202 | 2 | 0,000101 | 0,2036 | 0,816523 |  |
| G*T | 0,007877 | 2 | 0,003938 | 7,9520 | 0,001040 |  |
| N*T | 0,000748 | 1 | 0,000748 | 1,5109 | 0,224987 |  |
| G*W | 0,012726 | 2 | 0,006363 | 12,8475 | 0,000034 |  |
| N*W | 0,000159 | 1 | 0,000159 | 0,3213 | 0,573468 |  |
| T*W | 0,009311 | 1 | 0,009311 | 18,7996 | 0,000074 |  |
| G*N*T | 0,001130 | 2 | 0,000565 | 1,1406 | 0,328126 |  |
| G*N*W | 0,004261 | 2 | 0,002130 | 4,3016 | 0,019125 |  |
| G*T*W | 0,007877 | 2 | 0,003938 | 7,9520 | 0,001040 |  |
| N*T*W | 0,000748 | 1 | 0,000748 | 1,5109 | 0,224987 |  |
| G*N*T*W | 0,001130 | 2 | 0,000565 | 1,1406 | 0,328126 |  |
| Error | 0,023773 | 48 | 0,000495 |  |  |  |
|  |  |  |  |  |  |  |
| A+Z+V | SS | Tegr FreeTom | MS | F | p |  |
| Intercept | 6,232308 | 1 | 6,232308 | 443,7833 | 0,000000 |  |
| G+ATAR4:AW18 | 0,153091 | 2 | 0,076545 | 5,4506 | 0,007359 |  |
| N | 0,085358 | 1 | 0,085358 | 6,0781 | 0,017314 |  |
| T | 0,000102 | 1 | 0,000102 | 0,0073 | 0,932392 |  |
| W | 0,248015 | 1 | 0,248015 | 17,6604 | 0,000114 |  |
| G*N | 0,069799 | 2 | 0,034899 | 2,4851 | 0,093981 |  |
| G*T | 0,004760 | 2 | 0,002380 | 0,1695 | 0,844621 |  |
| N*T | 0,001715 | 1 | 0,001715 | 0,1221 | 0,728287 |  |
| G*W | 0,023059 | 2 | 0,011529 | 0,8210 | 0,446087 |  |
| N*W | 0,041652 | 1 | 0,041652 | 2,9659 | 0,091474 |  |
| T*W | 0,000102 | 1 | 0,000102 | 0,0073 | 0,932392 |  |
| G*N*T | 0,004915 | 2 | 0,002457 | 0,1750 | 0,840001 |  |
| G*N*W | 0,027687 | 2 | 0,013843 | 0,9857 | 0,380589 |  |
| G*T*W | 0,004760 | 2 | 0,002380 | 0,1695 | 0,844621 |  |
| N*T*W | 0,001715 | 1 | 0,001715 | 0,1221 | 0,728287 |  |
| G*N*T*W | 0,004915 | 2 | 0,002457 | 0,1750 | 0,840001 |  |
| Error | 0,674092 | 48 | 0,014044 |  |  |  |
|  |  |  |  |  |  |  |
| DEPS | SS | Tegr FreeTom | MS | F | p |  |
| Intercept | 4,270200 | 1 | 4,270200 | 1327,987 | 0,000000 |  |
| G | 0,007452 | 2 | 0,003726 | 1,159 | 0,322523 |  |
| N | 0,202305 | 1 | 0,202305 | 62,915 | 0,000000 |  |
| T | 0,174831 | 1 | 0,174831 | 54,370 | 0,000000 |  |
| W | 0,518356 | 1 | 0,518356 | 161,203 | 0,000000 |  |
| G*N | 0,022059 | 2 | 0,011029 | 3,430 | 0,040516 |  |
| G*T | 0,033100 | 2 | 0,016550 | 5,147 | 0,009437 |  |
| N*T | 0,006400 | 1 | 0,006400 | 1,990 | 0,164753 |  |
| G*W | 0,055267 | 2 | 0,027634 | 8,594 | 0,000645 |  |
| N*W | 0,027959 | 1 | 0,027959 | 8,695 | 0,004918 |  |
| T*W | 0,174831 | 1 | 0,174831 | 54,370 | 0,000000 |  |
| G*N*T | 0,011103 | 2 | 0,005552 | 1,727 | 0,188765 |  |
| G*N*W | 0,079892 | 2 | 0,039946 | 12,423 | 0,000045 |  |
| G*T*W | 0,033100 | 2 | 0,016550 | 5,147 | 0,009437 |  |
| N*T*W | 0,006400 | 1 | 0,006400 | 1,990 | 0,164753 |  |
| G*N*T*W | 0,011103 | 2 | 0,005552 | 1,727 | 0,188765 |  |
| Error | 0,154346 | 48 | 0,003216 |  |  |  |
|  |  |  |  |  |  |  |
| Neoxanthin | SS | Tegr FreeTom | MS | F | p |  |
| Intercept | 0,932286 | 1 | 0,932286 | 234,9163 | 0,000000 |  |
| G | 0,102623 | 2 | 0,051311 | 12,9294 | 0,000032 |  |
| N | 0,108518 | 1 | 0,108518 | 27,3442 | 0,000004 |  |
| T | 0,000545 | 1 | 0,000545 | 0,1372 | 0,712692 |  |
| W | 0,147521 | 1 | 0,147521 | 37,1722 | 0,000000 |  |
| G*N | 0,022988 | 2 | 0,011494 | 2,8962 | 0,064934 |  |
| G*T | 0,003036 | 2 | 0,001518 | 0,3825 | 0,684207 |  |
| N*T | 0,000028 | 1 | 0,000028 | 0,0071 | 0,932996 |  |
| G*W | 0,009017 | 2 | 0,004508 | 1,1360 | 0,329582 |  |
| N*W | 0,046452 | 1 | 0,046452 | 11,7049 | 0,001282 |  |
| T*W | 0,000545 | 1 | 0,000545 | 0,1372 | 0,712692 |  |
| G*N*T | 0,000625 | 2 | 0,000313 | 0,0788 | 0,924359 |  |
| G*N*W | 0,010349 | 2 | 0,005174 | 1,3038 | 0,280935 |  |
| G*T*W | 0,003036 | 2 | 0,001518 | 0,3825 | 0,684207 |  |
| N*T*W | 0,000028 | 1 | 0,000028 | 0,0071 | 0,932996 |  |
| G*N*T*W | 0,000625 | 2 | 0,000313 | 0,0788 | 0,924359 |  |
| Error | 0,190492 | 48 | 0,003969 |  |  |  |
|  |  |  |  |  |  |  |
| Lutein | SS | Tegr FreeTom | MS | F | p |  |
| Intercept | 8,499862 | 1 | 8,499862 | 340,5621 | 0,000000 |  |
| G | 0,681171 | 2 | 0,340586 | 13,6462 | 0,000020 |  |
| N | 0,611026 | 1 | 0,611026 | 24,4818 | 0,000010 |  |
| T | 0,004949 | 1 | 0,004949 | 0,1983 | 0,658102 |  |
| W | 0,850232 | 1 | 0,850232 | 34,0661 | 0,000000 |  |
| G*N | 0,110237 | 2 | 0,055118 | 2,2084 | 0,120918 |  |
| G*T | 0,011924 | 2 | 0,005962 | 0,2389 | 0,788441 |  |
| N*T | 0,000512 | 1 | 0,000512 | 0,0205 | 0,886705 |  |
| G*W | 0,076941 | 2 | 0,038470 | 1,5414 | 0,224493 |  |
| N*W | 0,178174 | 1 | 0,178174 | 7,1388 | 0,010271 |  |
| T*W | 0,004949 | 1 | 0,004949 | 0,1983 | 0,658102 |  |
| G*N*T | 0,007077 | 2 | 0,003539 | 0,1418 | 0,868176 |  |
| G*N*W | 0,069918 | 2 | 0,034959 | 1,4007 | 0,256318 |  |
| G*T*W | 0,011924 | 2 | 0,005962 | 0,2389 | 0,788441 |  |
| N*T*W | 0,000512 | 1 | 0,000512 | 0,0205 | 0,886705 |  |
| G*N*T*W | 0,007077 | 2 | 0,003539 | 0,1418 | 0,868176 |  |
| Error | 1,198000 | 48 | 0,024958 |  |  |  |
|  |  |  |  |  |  |  |
| β-carotene | SS | Tegr FreeTom | MS | F | p |  |
| Intercept | 2,251764 | 1 | 2,251764 | 228,6716 | 0,000000 |  |
| G | 0,173039 | 2 | 0,086520 | 8,7863 | 0,000560 |  |
| N | 0,251530 | 1 | 0,251530 | 25,5434 | 0,000007 |  |
| T | 0,000464 | 1 | 0,000464 | 0,0471 | 0,829083 |  |
| W | 0,285527 | 1 | 0,285527 | 28,9959 | 0,000002 |  |
| G*N | 0,061799 | 2 | 0,030899 | 3,1379 | 0,052389 |  |
| G*T | 0,010397 | 2 | 0,005199 | 0,5279 | 0,593207 |  |
| N*T | 0,000190 | 1 | 0,000190 | 0,0193 | 0,890131 |  |
| G*W | 0,017245 | 2 | 0,008623 | 0,8757 | 0,423138 |  |
| N*W | 0,109507 | 1 | 0,109507 | 11,1207 | 0,001652 |  |
| T*W | 0,000464 | 1 | 0,000464 | 0,0471 | 0,829083 |  |
| G*N*T | 0,002470 | 2 | 0,001235 | 0,1254 | 0,882432 |  |
| G*N*W | 0,026275 | 2 | 0,013138 | 1,3342 | 0,272968 |  |
| G*T*W | 0,010397 | 2 | 0,005199 | 0,5279 | 0,593207 |  |
| N*T*W | 0,000190 | 1 | 0,000190 | 0,0193 | 0,890131 |  |
| G*N*T*W | 0,002470 | 2 | 0,001235 | 0,1254 | 0,882432 |  |
| Error | 0,472663 | 48 | 0,009847 |  |  |  |
|  |  |  |  |  |  |  |
| Betacyanins | SS | Tegr FreeTom | MS | F | p |  |
| Intercept | 1026,067 | 1 | 1026,067 | 1151,022 | 0,000000 |  |
| G | 922,929 | 2 | 461,465 | 517,662 | 0,000000 |  |
| N | 1,149 | 1 | 1,149 | 1,289 | 0,261788 |  |
| T | 45,877 | 1 | 45,877 | 51,464 | 0,000000 |  |
| W | 317,563 | 1 | 317,563 | 356,236 | 0,000000 |  |
| G*N | 10,416 | 2 | 5,208 | 5,842 | 0,005359 |  |
| G*T | 63,876 | 2 | 31,938 | 35,827 | 0,000000 |  |
| N*T | 13,967 | 1 | 13,967 | 15,668 | 0,000249 |  |
| G*W | 510,132 | 2 | 255,066 | 286,128 | 0,000000 |  |
| N*W | 9,361 | 1 | 9,361 | 10,501 | 0,002170 |  |
| T*W | 45,877 | 1 | 45,877 | 51,464 | 0,000000 |  |
| G*N*T | 33,323 | 2 | 16,661 | 18,690 | 0,000001 |  |
| G*N*W | 49,957 | 2 | 24,979 | 28,020 | 0,000000 |  |
| G*T*W | 63,876 | 2 | 31,938 | 35,827 | 0,000000 |  |
| N*T*W | 13,967 | 1 | 13,967 | 15,668 | 0,000249 |  |
| G*N*T*W | 33,323 | 2 | 16,661 | 18,690 | 0,000001 |  |
| Error | 42,789 | 48 | 0,891 |  |  |  |
|  |  |  |  |  |  |  |
|  |  |  |  |  |  |  |
